# Estimation of heterosis and combining ability under low soil phosphorus condition in rice (*Oryza sativa* L.)

**DOI:** 10.1038/s41598-025-20344-8

**Published:** 2025-10-21

**Authors:** N. Madhusudan, P. Senguttuvel, R. M. Sundaram, M. S. Anantha, R. Gobinath, A. S. Hari Prasad, D. Subrahmanyam, K. V. RadhaKrishna

**Affiliations:** 1 Crop Improvement Section, ICAR-Indian Institute of Rice Research (ICAR-IIRR), Hyderabad, 500030 India; 2 Crop Production Section, ICAR-Indian Institute of Rice Research (ICAR-IIRR), Hyderabad, 500030 India; 3 Department of Genetics and Plant Breeding, Prof Jayashankar Telangana State Agricultural University (PJTSAU), Hyderabad, 500030 India

**Keywords:** Rice, Hybrids, Phosphorus, Heterosis, Combining ability, Physiology, Plant sciences

## Abstract

Exploiting yield heterosis under low soil phosphorus (P) inputs is a possible strategy for enhancing rice productivity and resource use efficiency in the changing climate scenario. In the present study, Line (L) × Tester (T) analysis was conducted with five restorers, three maintainer lines and 15 derived hybrids. The hybrids and parents were evaluated for two wet seasons (*Kharif*- 2017 and 2019) across the graded levels of applied P (low—P20 kg ha^−1^; Moderate—P40 kg ha^−1^; Normal—P60 kgha^−1^). Pooled analysis across years revealed highly significant variances in the interaction of the General Combining Ability (GCA) of parents and the Specific Combining Ability (SCA) of hybrids with a predominance of non-additive genetic variance. Testers contributed higher variation for the key traits under graded P conditions. Among the parental lines, CRMS32B showed strong GCA effects for single plant yield (SPY) and number of productive tillers per plant (NPTP), while IR79156B was promising for spikelet fertility percentage (SFP). Among testers, ATR305 and TCP795 exhibited significant GCA effects for SPY, SFP and NPTP and early flowering. The Hybrids, H14 (CRMS32A × ATR226) showed the highest SCA effects for NPTP, SPY and SFP, while H10 (IR79156A × TCP795) exhibited significant SCA effect for earliness. Compared to standard check variety Kasalath, the hybrids demonstrated a 120 to 200% heterosis gain across the graded P levels for the key traits. AMMI analysis identified hybrids, H3 (APMS6A × ATR305), H13 (CRMS32A × ATR305) and H11 (CRMS32A × AYT21) as highly stable performers across graded P levels, while H14 (CRMS32A × ATR226) with moderate stability. The present findings highlight the breeding rice hybrids suited for low P conditions to improve phosphorus use efficiency and ensure yield stability under resource-constrained environments.

**Supplementary Information:**

The online version contains supplementary material available at 10.1038/s41598-025-20344-8.

## Introduction

Rice (*Oryza sativa* L.) is a staple cereal for over half of the global population, ranks second in global cereal cultivation with 162 million hectares and 782 million metric tonnes of paddy produced annually, with china and India together contributing over 50 percent of the world’s total output (FAO, 2023). In 2022, India produced 135.75 million metric tonnes of rice from 47.83 million hectares, with an average yield of 2838 kg ha^−1^ (Indiastat, 2022–23). Despite two–threefold significant yield gains achieved since green revolution through adoption of high-yielding varieties and improved agronomic practices, yield stagnation in inbred lines has emerged as a major bottleneck in sustaining the pace of production growth. To address this, hybrid rice technology offers a promising approach, capable of exploiting yield heterosis to achieve significantly higher yields and enhance productivity. During the wet season (*Kharif* 2018–2024), approximately 3–4 million hectares (6–8% of the total 44 million hectares) were dedicated to hybrid rice cultivation, primarily in the eastern states such as Uttar Pradesh, Jharkhand, Chhattisgarh, Odisha and Bihar. This cultivation contributed an additional 4–5 million tonnes to India’s total rice production^1^ (http://aicrip-intranet.in 2024). The exploitation of rice heterosis has resulted in yield improvements of 15–20% over the best pure lines^2^.

Rice production is severely hampered by a range of biotic and abiotic stresses including nutrient deficiencies. Among these, Phosphorus (P) deficiency is a major abiotic limitation and it is a crucial macronutrient for plant growth but its availability in soil is limited due to interactions with soil components. In India, 49.3% of soils are low in available P, compared to a global estimate of 40% P-deficient cultivated land^3,4^. Globally, only about 12.6% of applied inorganic P fertilizer is utilized by plants, while 67.2% is retained in the soil and 4.4% is lost through leaching^5^. A significant portion of applied P becomes unavailable due to strong fixation with iron, aluminium, calcium, or magnesium compounds in soil reduce availability, leading to stunted growth^5^. To overcome low P conditions, rice plants have evolved multiple physiological and biochemical adaptations such as modification of root architecture by increased root length, density of root hairs, root diameter, and biomass, as well as proton extrusion to acidify the rhizosphere, thereby increasing P solubility. Additionally, they also exude organic acids, phenolics, mucilage, and enzymes (e.g., phosphatases, phytases) to mobilize bound P. Internal P remobilization from shoots to roots and older to younger leaves helps maintain P homeostasis^6^. Phosphorus use efficiency (PUE) is indicated by higher biomass or yield per unit of available P. Some rice genotypes show superior adaptation under P deficiency, with better biomass, photosynthesis, and antioxidant activity. In contrast, P deficiency typically causes delayed flowering, reduced height and tillers, weak stems, shorter roots and panicles, poor grain filling, and lower yields^7,8,9,10^.

Genetic variation for low P soil conditions in rice cultivars^7,11^ provides breeders to select or improve parental lines for hybrid development for low P soil conditions^8,9^ Harnessing heterosis using robust parental lines with favorable traits such as robust root architecture (root length, volume, biomass), shoot biomass, and enhanced P mobilization can improve P uptake, internal remobilization, and P use efficiency (PUE) for higher seed yield under low P conditions. However, relying solely on parental lines performance may lead to poor offspring/hybrids with low P uptake and PUE, resulting in stunted growth and reduced yield^12,10^. Hence, the genetic potential of parents, assessed through general and specific combining abilities, is crucial for successful heterosis breeding and the development of superior hybrids^13^. Successful hybrid development depends on the performance of desired traits with optimal combining ability, nature, and magnitude of gene action in various genetic backgrounds^14,15^. Line × tester analysis^16^ is an effective method for determining gene action and combining ability for quantitative traits. Line × Tester analysis of quantitative traits informs appropriate selection criteria and breeding strategies^17^. The presence of dominant and additive gene actions in a population facilitates the genetic improvement of traits. The degree of heterosis response varies with environmental conditions^18,19^. Keeping view of constraints in addressing food security through varietal improvement for unfavorable ecologies, the present study focused on identifying the best combining parents and stable hybrid heterosis for yield and its component traits in response to graded levels of applied phosphorus.

## Results

### ANOVA

The scope of cross × year interactions can be assessed by comparing the magnitude of the mean squares of cross × year interactions with those of the crosses. In our study, for all traits considered, the mean squares of cross × year interactions were consistently found to be non-significant across the graded levels of applied P. This indicates that variations observed in crop yield across years of cultivation do not have a significant impact. However, for all traits across the P levels, the mean squares of crosses were notably larger than those of the cross × year interaction mean squares. This suggests that the differences in performance among different crosses were more pronounced than any potential variations attributed to different years of cultivation. A significant difference (*P* < 0.05) in the mean sum of squares was observed for all genotypes (parents and hybrids), as well as for hybrids, across increasing levels of applied phosphorus. For most traits, the magnitude of variation decreased with higher P levels, except for the number of productive tillers, which showed an inverse trend.

### Performance of parents and hybrids across the graded levels of applied P

**Fig. 1:**
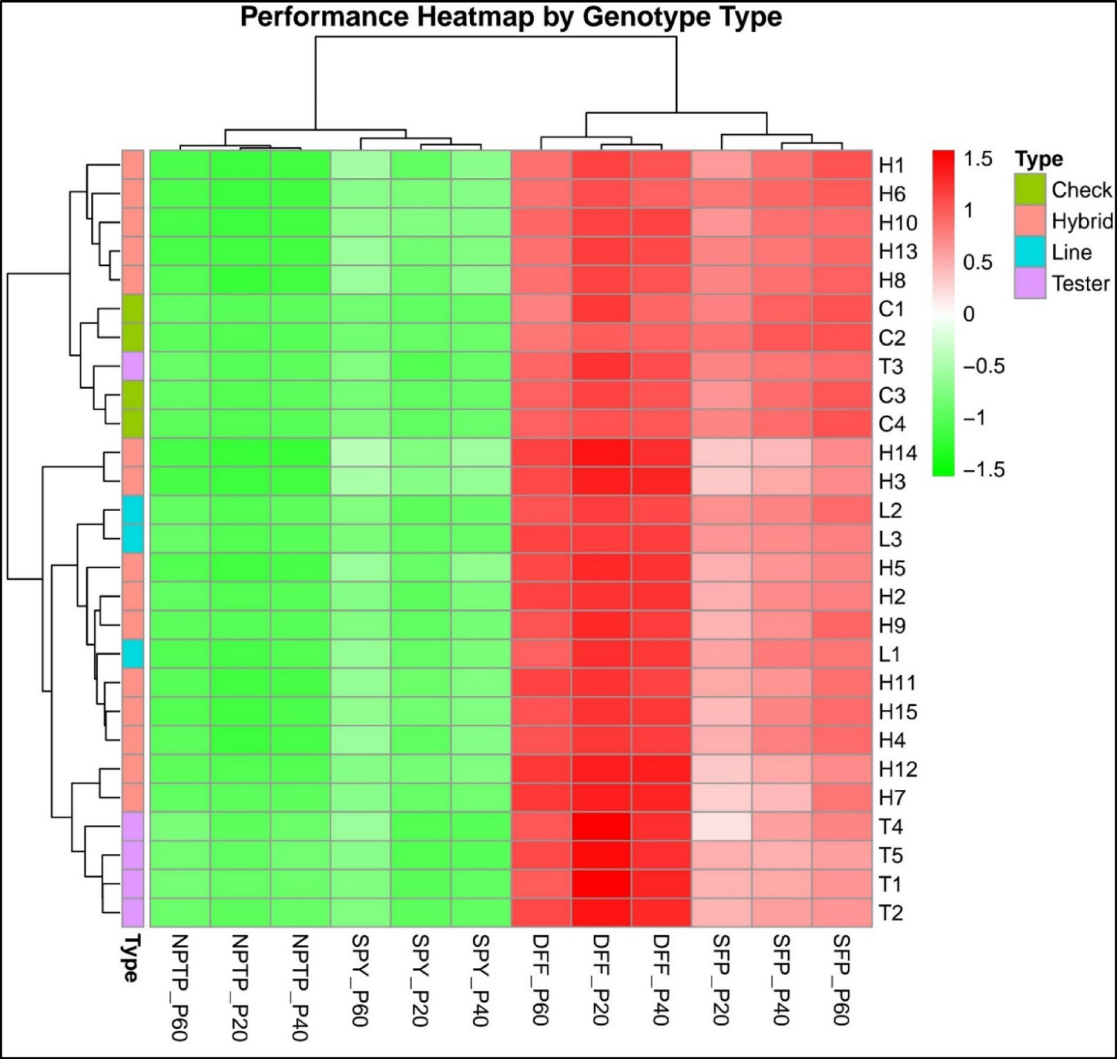
Hybrid, parental and checks performance per se for DFF—Days to 50% flowering, NPTP—Number of productive tillers per plant, SFP—Spikelet fertility percentage, SPY—Single plant yield (grams) across the graded level of applied P (20,40 and 60 kg ha^−1^).

Lines, testers, and hybrids showed significant variation for all studied traits (Table 1; Fig. 1). Flowering time decreased (from late to early) with increasing levels of applied phosphorus, while all other traits exhibited improved performance with higher phosphorus levels. Particularly, in terms of DFF, the hybrid H1 demonstrated earlier flowering compared to the line (IR79156B), tester (ATR305) and check variety (Rasi). The NPTP was highest in the line-IR79156B, tester-AYT21, check variety—Kasalath, and hybrid—H10 across graded levels of applied P. SFP was highest in the hybrids compared to the lines and tester. In addition, the hybrid—H14 exhibited the highest yield across all P levels compared to the lines and testers.

**Table 1:** Mean performance of lines, testers, hybrids and checks used in L × T mating design for DFF, NPTP, SFP and SPY in the graded level of applied P (20, 40 and 60 kg ha-1) ^20,40a −1^ environments. DFF—Days to 50% flowering; NPTP—Number of productive tillers per plant; SFP—Spikelet fertility percentage; SPY—Single plant yield (g); L1 to L4—Lines; T1 to T4—Testers; H1 to H15—Hybrids; C1 to C4—Checks; P20—P 20 kg ha^−1^; P40—P 40 kg ha^−1^; P60—P 60 kg ha^−1^.

Entry	DFF			NPTP			SFP			SPY		
	P20	P40	P60	P20	P40	P60	P20	P40	P60	P20	P40	P60
L1	106	103	93	6	8	9	75.28	85.84	88.15	13.10	18.51	25.36
L2	100	98	95	7	9	11	79.42	82.03	88.23	10.05	12.66	18.72
L3	105	105	103	4	6	10	80.55	83.62	85.91	8.42	9.53	15.65
T1	117	108	94	11	12	15	69.90	72.78	78.19	6.80	9.80	17.44
T2	113	107	99	8	11	13	70.25	76.25	78.98	8.37	9.90	17.90
T3	97	91	85	8	10	12	77.26	80.71	83.19	6.76	12.43	17.67
T4	116	104	93	8	12	15	56.56	74.19	80.33	4.90	6.24	23.53
T5	114	105	98	9	13	13	70.23	71.29	74.31	4.36	6.19	20.05
H1	91	88	81	6	7	9	71.00	80.40	87.74	13.02	22.41	29.06
H2	98	97	94	6	8	10	67.74	76.03	78.41	9.24	16.15	18.70
H3	110	108	99	5	6	8	67.43	74.55	81.78	22.62	26.93	31.94
H4	95	93	89	6	8	12	67.38	77.71	82.86	14.13	22.05	26.47
H5	97	94	90	7	8	10	67.01	72.84	75.51	15.45	23.74	27.07
H6	94	89	85	7	8	10	83.95	87.96	89.33	20.97	23.04	25.04
H7	100	99	94	8	9	10	58.61	63.95	79.17	10.86	13.17	19.23
H8	97	93	86	6	9	12	80.71	85.42	88.92	17.86	25.43	29.66
H9	98	93	88	7	8	9	64.57	73.59	83.17	9.56	14.63	17.35
H10	99	99	90	9	11	12	79.08	87.73	88.91	24.65	26.54	28.38
H11	94	90	90	7	8	11	66.81	71.97	80.14	15.99	20.63	24.95
H12	98	98	92	7	8	10	58.84	66.41	73.13	15.51	18.04	19.54
H13	97	94	85	8	9	10	79.94	84.33	87.35	19.08	23.40	28.35
H14	122	116	110	7	8	12	73.94	78.81	90.38	27.14	35.41	41.72
H15	97	95	90	7	9	10	66.11	77.86	83.31	17.71	20.64	23.78
C1	97	86	79	6	7	8	79.29	87.21	91.22	7.66	10.03	12.76
C2	88	86	81	6	7	8	82.79	88.22	90.33	10.40	11.15	13.61
C3	96	93	89	5	8	9	75.09	86.46	90.93	8.67	10.21	13.37
C4	94	92	89	5	6	8	80.18	86.73	93.35	9.34	12.24	15.42
Mean (Lines)	103	102	97	6	8	10	78.42	83.83	87.43	10.52	13.57	19.91
Mean (Testers)	111	103	94	9	12	14	68.84	75.04	79.00	6.24	8.91	19.32
Mean (Parents)	108	103	95	7	10	12	72.43	78.34	82.16	7.85	10.66	19.54
Mean (Crosses)	99	96	91	7	8	10	70.21	77.30	83.34	16.92	22.15	26.08
Mean (Total)	102	98	92	7	9	11	70.98	77.66	82.93	13.76	18.15	23.81

The importance of variance sources is indicated by the relative magnitude of variance components, with the SCA variance component being relatively significant at *P* < 0.05 and higher than the GCA variance for all traits, indicating the non-additive control of genetic variance (Table 2). Furthermore, there was a non-significant interaction of variance due to SCA with year for all the studied traits. Under low phosphorus (P) conditions, non-additive genetic variance was higher for flowering time, spikelet fertility percentage, and single plant yield; whereas it was lower under normal P conditions. In contrast, productive tillers showed the opposite trend, with higher non-additive variance under normal P and lower under low P conditions. The percentage contribution of lines and testers to the variation in hybrids is indicated in Table 2, with testers contributing a higher proportion of variation to hybrids compared to lines across graded level of applied P.

**Table 2:** Mean squares and genetic components for DFF, NPTP, SFP and SPY in Line x Tester (including parents) mating design in graded levels of applied P (20,40 and 60 kg ha^−1^) conditions.

Source of variation	df	DFF			NPTP			SFP			SPY		
		P20	P40	P60	P20	P40	P60	P20	P40	P60	P20	P40	P60
Replication (R)	1	3.924	3.193	3.52	0.02	0.45	0.55	0.45	3.60	1.40	0.02	0.31	4.92
Year (Y)	1	32.88*	65.05*	22.82*	42.75*	58.08*	61.84*	88.36*	50.88*	20.06*	56.40*	58.47*	44.16*
Replication × Year	1	0.53	0.008	0.02	0.05	0.164	0.55	0.39	0.351	0.6	0.27	0.02	0.07
Treatment (T)	22	305.45*	217.96*	171.42*	7.83*	13.98*	13.94*	233.25*	169.02*	107.13*	160.55*	223.23*	152.73*
Parents (P)	7	235.91*	128.81*	113.17*	15.20*	22.89*	20.82*	236.61*	117.52*	102.12*	32.11*	63.32*	43.68*
Parents (Line)	2	40.58*	55.38*	118.08*	6.87*	6.64*	1.70	30.82*	14.67*	6.97*	22.59*	83.09*	98.47*
Parents(Testers)	4	271.55*	194.20*	123.43*	6.78*	6.62*	5.51*	226.80*	53.46*	42.01*	10.44*	28.66*	26.56*
Parents (L vs T)	1	484.01*	14.09*	62.35*	65.54*	120.47*	120.31*	687.44*	579.46*	532.84*	137.83*	162.40*	2.63
Parent vs Crosses	1	1800.16*	842.69*	367.77*	9.74*	76.29*	89.20*	103.12*	22.31*	29.05*	1718.83*	2754.32*	893.17*
Crosses	14	233.46*	217.91*	186.52*	4.00*	5.08*	5.12*	240.86*	205.25*	115.21*	113.46*	122.39*	154.36*
Line effect	2	92.85	77.91	121.72	7.48	13.3	3.81	156.46	89.21	111.09	88.28	46.99	74.59
Tester effect	4	252.89	239.13	183.79	5.69	4.06	1.38	366.9	307.19	178.39	119.04	167.7	207.82
Line × Tester effect	8	258.89*	242.29*	204.09*	2.29*	3.53*	7.32*	198.95*	183.30*	84.64*	116.97*	118.59*	147.58*
Year × Treat	22	0.70	0.69	0.40	0.62*	0.24	0.30	0.91	1.37	0.54	0.77	0.73	0.8
Year × Parents	7	0.50	0.96	0.25	0.44	0.08	0.47	0.08	0.44	0.17	0.08	0.28	0.33
Year × Parents (L)	2	0.58	0.05	0.08	0.32	0.05	0.47	0.05	0.24	0.12	0.10	0.68	0.32
Year × Parents (T)	4	0.55	1.45	0.38	0.60	0.08	0.28	0.01	0.64	0.19	0.09	0.07	0.42
Year × PAR (L vs T)	1	0.13	0.85	0.05	0.03	0.18	1.24	0.39	0.04	0.22	0.004	0.31	0.01
Year × Parent × Cross	1	0.47	0.64	0.15	5.29*	2.00*	1.10	5.46	0.038	0.01	0.11	0.10	0.65
Year × Crosses	14	0.81	0.56	0.5	0.38	0.19	0.16	1.00	1.93	0.76	1.16	0.99	1.05
Year × Line effect	2	1.85*	1.56*	0.32	1.88*	0.21	0.15	3.52*	6.45*	0.45	4.75*	1.87	0.52
Year × Tester effect	4	1.23	0.55	0.69	0.023	0.29	0.05	0.85	1.95	0.81	0.94	0.93	1.58
Year × Line × Tester effect	8	0.35	0.32	0.44	0.181	0.13	0.21	0.45	0.79	0.81	0.38	0.81	0.91
Error	44	1.89	1.51	0.90	0.282	0.38	0.57	3.84	2.37	1.10	1.32	1.01	3.40
Total	91	75.34	54.359	45.2	2.649	4.26	4.41	59.45	42.94	26.8	40.26	55.28	39.30
Genetic components													
σ^2^ GCA		10.68	9.83	9.49	0.4	0.52	0.14	16.1	12.2	8.99	6.37	6.63	8.56
σ^2^ SCA		64.23*	60.25*	50.80*	0.52*	0.80*	1.72*	48.71*	45.08*	20.92*	28.80*	29.32*	35.85*
σ^2^ GCA/σ^2^ SCA		0.17	0.16	0.19	0.77	0.65	0.08	0.33	0.27	0.43	0.22	0.23	0.24
σ^2^ Year		0.51*	1.03*	0.48*	0.33	0.61*	1.05*	0.75	0.63	0.25*	0.71	0.75*	0.43*
σ^2^ Year × GCA		− 0.06*	− 0.03	− 0.05	0.09	− 0.01	− 0.04	− 0.24*	0.15*	− 0.04	0.14*	0.01	− 0.39
σ^2^ Year × SCA		− 0.82	− 0.5	− 0.23	− 0.01*	− 0.09	− 0.11	− 1.82	− 1.09	− 0.08	− 0.69	− 0.25	− 1.64
σ^2^ Year × GCA/σ^2^ Year × SCA		0.07	0.06	0.22	− 9.00	0.11	0.36	0.13	− 0.14	0.5	− 0.20	− 0.04	0.24
Proportional contribution of Lines, Testers and Line × Tester in the mating design													
Line		5.68	5.11	9.32	26.70	37.43	10.64	9.28	6.21	13.77	11.12	5.49	6.90
Tester		30.95	31.35	28.15	40.65	22.82	7.69	43.52	42.76	44.24	29.98	39.15	38.47
Line × Tester		63.37	63.54	62.52	32.65	39.75	81.68	47.20	51.03	41.98	58.91	55.37	54.63

df—Degrees of freedom; Where DFF—Days to 50% flowering; NPTP—Number of productive tillers per plant; SFP- Spikelet fertility percentage, SPY- Single plant yield (g); L1 to L3—Lines; T1 to T5—Testers; H1 to H15—Hybrids. P20—P 20 kg ha^−1^; P40—P 40 kg ha^−1^; P60—P 60 kg ha^−1^; *P < 0.05.

### Combining ability studies

#### GCA effects on parents

**Fig. 2:**
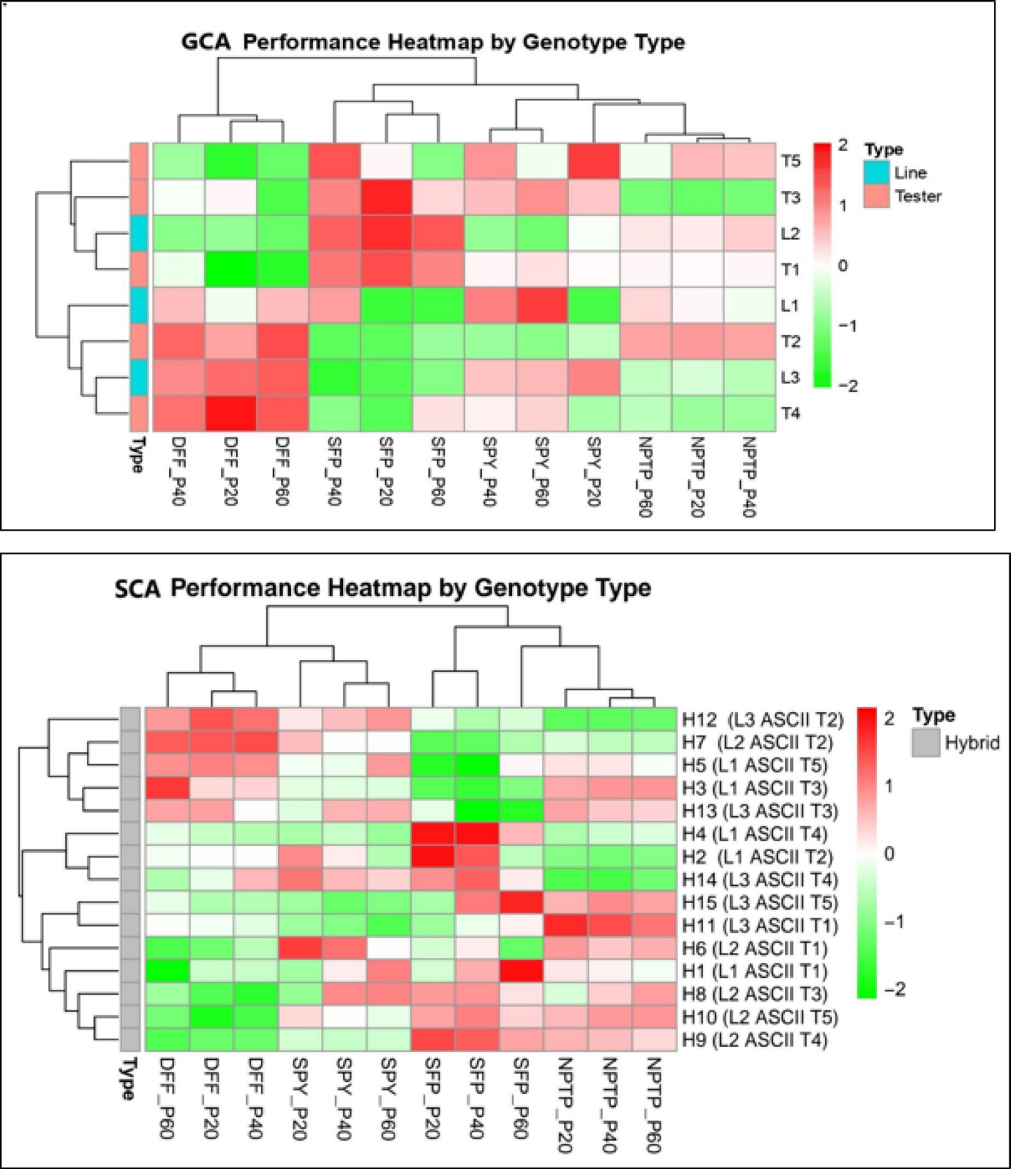
GCA and SCA performance of parental lines and hybrids for DFF—Days to 50% flowering, NPTP—Number of productive tillers per plant, SFP—Spikelet fertility percentage, SPY—Single plant yield (grams) across the graded level of applied P (20,40 and 60 kg ha^−1^).

The primary objective of the combining ability study was to identify parental lines suitable for hybrid rice improvement. In this context, evaluation of a parent’s GCA and crosses SCA effect play a critical role in producing superior hybrids. Estimates of GCA effects for the traits under study for eight parents are mentioned in Table 3 (Fig. 2; Figure S1, S2, S3). None of the parents exhibited consistently significant positive GCA effects across all the studied traits. Notably, line L2 (100, 98 and 95 days at 20, 40 and 60 kg ha^−1^ of applied P) and tester T1 (117, 108 and 94 days at 20, 40 and 60 kg ha^−1^ of applied P) displayed significant negative GCA effects across graded levels of applied P, indicating early crop maturation. Line-L2 (7, 9 and 11 at 20, 40 and 60 kg ha^−1^ of applied P) demonstrated a significant positive GCA effect across the graded levels of applied P for NPTP, while tester T5 exhibited significance positive at P20 and P40 kg ha^−1^ of 9 and 13 NPTP, respectively but displayed a negative GCA effect of at other level (13 NPTP). Lines—L2 for SFP (79.42, 82.03 and 88.23% at 20, 40 and 60 kg ha^−1^ of applied P) and L3 for SPY (8.42, 9.53 and 15.65 g at 20, 40 and 60 kg ha^−1^ of applied P) exhibited the highest significant positive GCA effects across graded levels of applied P. Additionally, tester T3 displayed the highest positive GCA effect among graded levels of applied P for both SFP (77.26, 80.71 and 83.19% at 20, 40 and 60 kg ha^−1^ of applied P) and SPY (6.76, 12.43 and 17.67 g at 20, 40 and 60 kg ha^−1^ of applied P). These results of GCA effect of parental lines with their per se performance can be utilized for development best recombinant pureline varieties.

**Table 3:** Estimation of relative general combining ability (GCA) effects of parents, relative specific combining ability (SCA) effects of crosses (Hybrids) for DFF, NPTP, SFP and SPY in Line × Tester (including parents) mating design in graded levels of applied P y conditions*.*

Entries		DFF			NPTP			SFP			SPY		
		P20	P40	P60	P20	P40	P60	P20	P40	P60	P20	P40	P60
Lines	L1	− 0.85*	− 0.33	− 0.33	− 0.71*	− 0.85*	− 0.49*	− 2.10*	− 0.10*	− 2.08*	− 2.03*	0.11	0.57
	L2	− 1.60*	− 1.79*	− 2.28*	0.32*	0.78*	0.34*	3.18*	2.43*	2.56*	− 0.14	− 1.58*	− 2.15*
	L3	2.45*	2.12*	2.62*	0.39*	0.07	0.16	− 1.08*	− 1.43*	− 0.48*	2.17*	1.48*	1.59*
	SE of Lines	0.32	0.26	0.21	0.1	0.13	0.15	0.42	0.39	0.22	0.3	0.26	0.46
Testers	T1	− 6.32*	− 0.74*	− 5.33*	− 0.32*	− 0.23	− 0.18	3.72*	2.81*	2.40*	− 0.26	− 0.12	0.27
	T2	− 0.23	1.76*	2.67*	0.12	− 0.19	− 0.13	− 8.48*	− 8.51*	− 6.44*	− 5.05*	− 6.36*	− 6.93*
	T3	2.10*	1.85*	− 1.08*	− 0.68*	− 0.40*	− 0.40*	5.82*	4.13*	2.68*	2.93*	3.11*	3.90*
	T4	6.10*	4.27*	4.83*	− 0.25	− 0.22	0.37	− 1.58*	− 0.6	2.13*	0.03	1.88*	2.43*
	T5	− 1.65*	− 0.48	− 1.08*	1.13*	1.03*	0.34	0.53	2.17*	− 0.77*	2.35*	1.49*	0.33
	SE of testers	0.41	0.33	0.27	0.13	0.16	0.19	0.58	0.5	0.28	0.38	0.33	0.59
Hybrids	H1 (L1 × T1)	− 0.98	− 1	− 4.42*	0.38	0.19	− 0.29	− 0.83	1.29	4.08*	− 1.61*	0.28	2.15*
	H2 (L1 × T2)	2.27*	2.20*	2.03*	− 0.07	− 0.35	0.01	6.86*	5.42*	1.03*	4.45*	2.60*	0.84
	H3 (L1 × T3)	− 1.28	− 1.20*	2.38*	− 0.29	0.16	0.27	− 6.03*	− 6.71*	− 5.12*	− 2.84*	− 2.88*	− 2.99*
	H4 (L1 × T4)	0.18	− 0.41	1.08*	− 0.51*	0.38	0.75*	8.11*	8.23*	3.59*	− 0.6	0.25	− 1.03
	H5 (L1 × T5)	2.93*	2.52*	2.53*	0.34	0.08	− 0.68*	− 6.30*	− 7.27*	− 0.29	− 0.87	− 1.03	2.22*
	H6 (L2 × T1)	− 3.12*	− 2.11*	− 3.62*	0.17	− 0.46	− 0.08	− 1.81	− 0.96	− 3.30*	1.47*	0.78	− 1.2
	H7 (L2 × T2)	9.60*	10.25*	9.33*	− 0.38	− 1.46*	− 1.41*	− 6.50*	− 5.89*	− 2.16*	4.80*	1.57*	1.39
	H8 (L2 × T3)	− 2.90*	− 3.55*	− 1.72*	− 0.74*	0.69*	1.43*	1.507	1.56	0.35	− 1.86*	1.76*	1.83
	H9 (L2 × T4)	− 6.70*	− 6.70*	− 7.62*	1.12*	0.77*	− 0.03	4.99*	4.33*	1.81*	− 2.94*	− 3.33*	− 3.22*
	H10 (L2 × T5)	− 9.15*	− 7.67*	− 6.33*	0.26	1.17*	1.41*	0.85	2.00*	− 0.53	− 0.79	− 2.09*	− 2.61*
	H11 (L3 × T1)	− 5.40*	− 5.71*	− 5.13*	− 0.33	− 1.10*	− 2.08*	− 7.23*	− 5.54*	− 4.86*	− 7.24*	− 7.82*	− 9.01*
	H12 (L3 × T2)	14.55*	13.38*	11.47*	0.07	− 0.07	0.67	6.39*	3.54*	5.39*	8.03*	9.91*	11.62*
	H13 (L3 × T3)	0.35	− 1.17	0.33	0.26	− 0.28	− 0.47	− 1.63	− 5.64*	− 4.99*	− 1.79*	− 0.01	0.09
	H14 (L3 × T4)	3.10*	4.54*	2.28*	0.80*	0.69*	1.31*	5.17*	5.83*	3.77*	5.52*	4.49*	4.12*
	H15 (L3 × T5)	− 3.45*	− 3.37*	− 2.62*	− 1.06*	− 0.41	− 0.84*	− 3.54*	− 0.19	1.22*	− 3.73*	− 4.48*	− 4.21*
	SE of Hybrids	0.71	0.58	0.47	0.23	0.28	0.33	1.01	0.86	0.49	0.66	0.57	1.02

Where DFF—Days to 50% flowering; NPTP- Number of productive tillers per plant; SFP- Spikelet fertility percentage; SPY—Single plant yield (g); L1 to L3—Lines; T1 to T5—Testers; H1 to H15—Hybrids. P20—P 20 kg ha^−1^ ; P40—P 40 kg ha^−1^ ; P60—P 60 kg ha^−1^ ; *P < 0.05.

#### SCA effect of crosses

SCA effects of crosses varied from negative to positive for the studied traits under graded levels of applied P are shown in Table 3 (Fig. 2; Figure S4, S5, S6). Hybrid H10 displayed a significantly increasing negative SCA effect at *P* < 0.05 from normal to low concentrations of applied P for early flowering with significant positive standard heterosis at *P* < 0.05 (2.59, 14.20 and 12.93 percent at 20, 40, 60 kg ha^−1^ applied P). Most of the hybrids for NPTP and SFP exhibited a decreasing SCA effect from normal to low applied P levels in which H9 reported high significant positive SCA effect at *P* < 0.05(for NPTP) in low and medium applied P with positive standard heterosis of 2.55 percent and significant positive standard heterosis at *P* < 0.05 of 16.32 percent respectively, but negative non-significant in normal applied P with 15.13 percent significant positive standard heterosis. Hybrid H4 demonstrated the highest significant positive SCA effect (*P* < 0.05) for SFP at moderate levels of applied P (40 kg ha^−1^) with negative significant standard heterosis 10.90 percent, followed by low levels of applied P (20 kg ha^−1^) with negative significant (*P* < 0.05) standard heterosis of 15.02 percent. SPY exhibited a mixed response in SCA effects across graded applied P but a significant positive high SCA effect in hybrid H12 from low to high levels of applied P with positive significant standard heterosis of 102.38, 79.86 and 53.15 percent at applied P of 20, 40 and 60 kg ha^−1^. High SCA effect with high standard heterosis may be utilized in development of hybrid cultivars. However, in our study no single hybrid found higher performance across traits under graded level of applied P.

#### Heterosis

Three types of heterosis, namely Mid-Parent heterosis (MPH), Better Parent Heterosis (BPH) and Standard Heterosis (SH), were estimated to assess the performance of hybrid combinations across graded levels of applied P. MPH exhibited significant positive values in most of the hybrids for SPY, except for hybrid, which displayed H2 negative heterosis under P20 and P40 (Table 4). Conversely, hybrids showed significant negative MPH for early flowering, except for H3 and H14, which displayed significant positive heterosis across different levels of applied P. The H13 hybrid exhibited a significant positive MPH under low and moderate levels of applied P for NPTP. Significant positive MPH for SFP was observed in hybrids—H6 and H10 across the graded applied P (Figures S7, S8, S9). Findings of MPH help plant breeders to identify high additive effect at many loci in genome.

**Table 4:** Estimation of mid-parent heterosis (MPH) for DFF, NPTP, SFP and SPY of crosses (Hybrids) in graded levels of applied P (20,40 and 60 kg ha^−1^) conditions*.*

Entries	DFF			NPTP			SFP			SPY		
	P20	P40	P60	P20	P40	P60	P20	P40	P60	P20	P40	P60
H1 (L1 × T1)	− 18.20*	− 17.23*	− 13.44*	− 27.27*	− 28.00*	− 23.75*	− 2.19	1.37	5.49*	30.92*	58.36*	35.81*
H2 (L1 × T2)	− 10.07*	− 7.57*	− 1.96*	− 16.93*	− 21.95*	− 8.02*	− 6.91*	− 6.18*	− 6.17*	− 13.92	13.65*	− 13.54*
H3 (L1 × T3)	8.64*	11.23*	10.99*	− 29.04*	− 37.63*	− 26.86*	− 11.59*	− 10.48*	− 4.54*	127.80*	74.07*	48.46*
H4 (L1 × T4)	− 14.09*	− 10.82*	− 4.31*	− 12.74*	− 16.13*	− 5.46	2.22	− 2.88*	− 1.63*	56.97*	78.18*	8.29
H5 (L1 × T5)	− 11.62*	− 9.35*	− 5.91*	− 2.1	− 23.08*	− 15.38*	− 7.90*	− 7.28*	− 7.05*	76.95*	92.23*	19.22*
**Mean**	− 9.07	− 6.75	− 2.93	− 17.62	− 25.36	− 15.89	− 5.27	− 5.09	− 2.78	55.74	63.3	19.65
H6 (L2 × T1)	− 13.73*	− 13.24*	− 9.57*	− 21.87*	− 19.60*	− 18.54*	12.45*	13.64*	7.35*	148.93*	105.16*	38.45*
H7 (L2 × T2)	− 5.76*	− 3.57*	− 3.48*	7.91	− 10.83*	− 17.63*	− 21.69*	− 19.19*	− 5.30*	17.92	16.74*	5.03
H8 (L2 × T3)	− 1.65	− 1.59*	− 4.74*	− 21.35*	1.65	1.15	3.02	4.98*	3.75*	112.43*	102.71*	63.01*
H9 (L2 × T4)	− 9.03*	− 7.81*	− 6.13*	− 8.70*	− 24.63*	− 30.57*	− 5.02*	− 5.79*	− 1.32	27.91*	54.75*	− 17.89*
H10 (L2 × T5)	− 7.37*	− 2.60*	− 6.89*	15.89*	− 1.2	2.14	5.69*	14.45*	9.40*	242.06*	181.53*	46.39*
**Mean**	− 7.51	− 5.76	− 6.16	− 5.62	− 10.92	− 12.69	− 1.11	1.62	2.78	109.85	92.18	27
H11 (L3 × T1)	− 15.32*	− 15.63*	− 8.03*	− 11.12*	− 11.11*	− 14.40*	− 11.18*	− 7.97*	− 2.32*	110.25*	113.48*	50.76*
H12 (L3 × T2)	− 9.86*	− 7.44*	− 8.66*	28.46*	− 12.63*	− 10.18*	− 21.97*	− 16.92*	− 11.30*	84.78*	85.62*	16.52*
H13 (L3 × T3)	− 3.96*	− 4.35*	− 9.99*	26.22*	9.45*	− 8.92*	1.31	2.64*	3.31*	151.47*	113.16*	70.17*
H14 (L3 × T4)	10.51*	11.14*	11.88*	17.67*	− 10.75*	− 6.72*	7.86*	− 0.12	8.74*	307.62*	349.08*	112.94*
H15 (L3 × T5)	− 11.87*	− 9.68*	− 10.72*	10.06*	− 7.29	− 13.48*	− 12.30*	0.52	4.00*	177.17*	162.57*	33.22*
Mean	− 6.10	− 5.19	− 5.10	14.26	− 6.47	− 10.74	− 7.26	− 4.37	0.49	166.26	164.78	56.72
Total mean	− 7.56	− 5.9	− 4.73	− 2.99	− 14.25	− 13.11	− 4.55	− 2.61	0.16	110.62	106.75	34.46

Where DFF—Days to 50% flowering; NPTP—Number of productive tillers per plant, SFP—Spikelet fertility percentage, SPY—Single plant yield (g); L1 to L3—Lines; T1 to T5—Testers; H1 to H15—Hybrids. P20—P 20 kg ha^−1^; P40—P 40 kg ha^−1^; P60—P 60 kg ha^−1^; *P < 0.05.

Hybrid entries displayed significant negative BPH for flowering with a decreasing order of heterotic levels from low to normal levels of applied P (Table 5). Some hybrids for the NPTP recorded negative BPH, with an ascending order in their values across the graded levels of applied P, except for H7, which showed positive BPH under P20 but displayed negative values in both P40 and P60. Most hybrids exhibited negative BPH for SFP, with a descending order from normal to low levels of applied P, and some hybrids with positive BPH were H6 > H8 in P20; H6 > H10 > H8 > H13 in P40; and H14 > H13 > H6 > H8 > H10 in P60. SPY was positive in most of the studied hybrids and H14 recorded the highest BPH across the graded levels of applied P, peculiarly most of the hybrids reveal high BPH in moderate than low and normal applied P conditions. Hybrid with high BPH indicates dominance gene action at many loci in the genome.

**Table 5:** Estimation of better parent heterosis (BPH) for DFF, NPTP, SFP and SPY of crosses (Hybrids) in graded levels of applied P (20,40 and 60 kg ha^−1^) conditions.

Entries	DFF			NPTP			SFP			SPY		
	P20	P40	P60	P20	P40	P60	P20	P40	P60	P20	P40	P60
H1 (L1 × T1)	− 22.22*	− 19.17*	− 13.90*	− 42.42*	− 40.37*	− 38.08*	− 5.68*	− 6.34*	− 0.47	− 0.57	21.10*	14.61*
H2 (L1 × T2)	− 13.05*	− 9.32*	− 5.29*	− 23.98*	− 33.10*	− 21.35*	− 10.01*	− 11.43*	− 11.05*	− 29.47*	− 12.77*	− 26.26*
H3 (L1 × T3)	4.27*	4.66*	6.49*	− 36.77*	− 42.65*	− 35.81*	− 12.73*	− 13.15*	− 7.22*	72.65*	45.48*	25.96*
H4 (L1 × T4)	− 18.06*	− 11.27*	− 4.57*	− 21.43*	− 29.26*	− 23.25*	− 10.49	− 9.48*	− 6.00*	7.84	19.14*	4.4
H5 (L1 × T5)	− 14.91*	− 10.02*	− 8.44*	− 17.38*	− 37.69*	− 28.06*	− 10.98*	− 15.14*	− 14.34*	17.92*	28.26*	6.74
Mean	− 12.79	− 9.02	− 5.14	− 28.4	− 36.61	− 29.31	− 9.98	− 11.11	− 7.82	13.67	20.24	5.09
H6 (L2 × T1)	− 20.09*	− 17.55*	− 10.05*	− 36.98*	− 31.65*	− 30.62*	5.71*	7.23*	1.24	108.63*	81.95*	33.72*
H7 (L2 × T2)	− 11.28*	− 7.95*	− 5.79*	1.03	− 21.44*	− 25.86*	− 26.21*	− 22.04*	− 10.27*	8.03	4.03	2.71
H8 (L2 × T3)	− 3.01*	− 4.87*	− 9.52*	− 28.35*	− 3.69	− 6.38	1.62	4.13*	0.79	77.62*	100.83*	58.43*
H9 (L2 × T4)	− 15.48*	− 10.79*	− 6.88*	− 15.93*	− 34.71*	− 40.89*	− 18.69*	− 10.30*	− 5.74*	− 4.87	15.52*	− 26.28*
H10 (L2 × T5)	− 13.16*	− 5.97*	− 8.44*	− 0.17	− 17.92*	− 8.61*	− 0.43	6.95*	0.77	145.21*	109.58*	41.55*
Mean	− 12.6	− 9.43	− 8.14	− 16.08	− 21.88	− 22.47	− 7.6	− 2.81	− 2.64	66.92	62.38	22.03
H11 (L3 × T1)	− 19.66*	− 17.09*	− 12.17*	− 38.36*	− 33.23*	− 30.06*	− 17.05*	− 13.93*	− 6.71*	90.02*	110.54*	43.01*
H12 (L3 × T2)	− 13.05*	− 8.62*	− 10.22*	− 0.4	− 32.44*	− 22.69*	− 26.95*	− 20.58*	− 14.87*	84.23*	82.08*	9.21
H13 (L3 × T3)	− 7.62*	− 10.53*	− 17.76*	− 4.15	− 10.13*	− 19.52*	− 0.76	0.85	1.68*	126.67*	88.29*	60.44*
H14 (L3 × T4)	5.16*	11.00*	6.57*	− 9.91*	− 31.92*	− 23.82*	− 8.21*	− 5.76*	5.21*	222.45*	271.66*	77.28*
H15 (L3 × T5)	− 15.35*	− 9.79*	− 12.90*	− 19.87*	− 31.64*	− 25.95*	− 17.92*	− 6.89*	− 3.02*	110.37*	116.58*	18.60*
Mean	− 10.1	− 7.01	− 9.3	− 14.54	− 27.87	− 24.41	− 14.18	− 9.26	− 3.54	126.75	133.83	41.71
Total mean	− 11.83	− 8.49	− 7.53	− 19.67	− 28.79	− 25.40	− 10.59	− 7.73	− 4.67	69.11	72.15	22.94

Where DFF—Days to 50% flowering; NPTP—Number of productive tillers per plant, SFP—Spikelet fertility percentage, SPY—Single plant yield (g); L1 to L3—Lines; T1 to T5—Testers; H1 to H15—Hybrids. P20—P 20 kg ha^−1^; P40—P 40 kg ha^−1^; P60—P 60 kg ha^−1^ ; *P < 0.05.

Standard Heterosis (SH) was highest for SPY exhibited by H14 across the three graded levels of applied P (Table 6). None of the hybrids exhibited concurrent SH for all the studied traits. For early flowering, hybrids—H1 > H6 > H11 > H4 showed negative heterosis under P20, with SH decreasing from normal to low levels of applied P. Except for four hybrids in P20 and one hybrid in P40 for NPTP, negative SH was noted; the remaining hybrids showed increasing positive SH across the graded levels of applied P, with H10 recording the highest SH. Hybrids exhibited a decrease in SFP from normal P to low P, with H6 > H10 > H13 displaying the highest positive SH in P20 and H6 > H10 showing positive SH in P40. H14 exhibited the highest SH, even though none of the hybrids displayed positive SH for SFP in P60. Hybrid with high BPH indicates dominance gene action at many loci in the genome.

**Table 6:** Estimation of standard heterosis (SH) for DFF, NPTP, SFP and SPY of crosses (Hybrids) in graded levels of applied P (20,40 and 60 kg ha^−1^) conditions.

Entries	DFF			NPTP			SFP			SPY		
	P20	P40	P60	P20	P40	P60	P20	P40	P60	P20	P40	P60
H1 (L1 × T1)	− 5.70*	1.45	1.58	− 3.61	11.17	20.48*	− 10.45*	− 7.81*	− 3.81*	69.98*	123.51*	127.76*
H2 (L1 × T2)	1.81	12.75*	18.61*	− 10.56*	14.63*	34.52*	− 14.56*	− 12.82*	− 14.04*	20.59	61.01*	46.53*
H3 (L1 × T3)	13.99*	25.22*	24.29*	− 21.04*	− 16.36*	3.23	− 14.96*	− 14.52*	− 10.34*	195.17*	168.51*	150.31*
H4 (L1 × T4)	− 1.3	7.25*	11.99*	− 4.16	26.02*	49.48*	− 15.02*	− 10.90*	− 9.16*	84.37*	119.90*	107.46*
H5 (L1 × T5)	0.52	9.28*	12.93*	17.35*	22.94*	24.74*	− 15.49*	− 16.48*	− 17.23*	101.60*	136.72*	112.13*
Mean	1.86	11.19	13.88	− 4.4	11.68	26.49	− 14.1	− 12.51	− 10.92	94.34	121.93	108.84
H6 (L2 × T1)	− 3.11*	3.48*	7.26*	5.49	27.42*	35.00*	5.89*	0.86	− 2.07*	173.70*	129.77*	96.20*
H7 (L2 × T2)	3.89*	14.46*	17.98*	18.88*	34.60*	26.81*	− 26.08*	− 26.68*	− 13.20*	41.73*	31.36*	50.71*
H8 (L2 × T3)	0.26	7.54*	7.89*	− 10.52*	40.47*	50.55*	1.79	− 2.06	− 2.51*	133.02*	153.60*	132.46*
H9 (L2 × T4)	1.81	7.83*	11.04*	2.55	16.32*	15.13*	− 18.56*	− 15.63*	− 8.83*	24.8	45.87*	35.95*
H10 (L2 × T5)	2.59*	14.20*	12.93*	41.80*	61.94*	58.45*	− 0.26	0.6	− 2.53*	221.70*	164.65*	122.41*
Mean	1.09	9.5	11.42	11.64	36.15	37.19	− 7.44	− 8.58	− 5.83	118.99	105.05	87.55
H11 (L3 × T1)	− 2.59*	4.06*	13.88*	3.18	24.48*	36.10*	− 15.73*	− 17.48*	− 12.14*	108.74*	105.71*	95.49*
H12 (L3 × T2)	1.81	13.62*	16.40*	17.19*	15.76*	32.23*	− 25.79*	− 23.86*	− 19.83*	102.38*	79.86*	53.15*
H13 (L3 × T3)	0.52	8.41*	6.62*	19.70*	31.06*	29.42*	0.82	− 3.31*	− 4.24*	149.00*	133.36*	122.18*
H14 (L3 × T4)	26.68*	34.49*	38.17*	9.89	21.29*	48.39*	− 6.74*	− 9.64*	− 0.92	254.23*	253.13*	226.94*
H15 (L3 × T5)	0	9.57*	12.93*	13.81*	34.86*	28.39*	− 16.61*	− 10.73*	− 8.67*	131.09*	105.78*	86.36*
Mean	5.28	14.03	17.6	12.75	25.49	34.91	− 12.81	− 13	− 9.16	149.09	135.57	116.82
Total mean	2.75	11.57	14.3	6.66	24.44	32.86	− 11.45	− 11.36	− 8.63	120.81	120.85	104.4

Where DFF—Days to 50% flowering; NPTP—Number of productive tillers per plant, SFP—Spikelet fertility percentage, SPY—Single plant yield (g); L1 to L3—Lines; T1 to T5—Testers; H1 to H15—Hybrids. P20—applied P 20 kg ha^−1^; P40—applied P 40 kg ha^−1^; P60—applied P 60 kg ha^−1^; *P < 0.05.

### Correlation between phenotype, heterosis and combining ability

The association between phenotype, heterosis and combining ability effects for the studied traits was observed across grades of applied phosphorus (Table S1). GCA did not have any significant correlation with SCA effects, SH, BPH, MPH and phenotype for all traits across the graded levels of applied P. SCA had positive significant (*P* < 0.05) correlations with SH for DFF in P60. GSCA (General sum of combining ability) had a significant (*P* < 0.01) positive association with SCA, SH, MPH and BPH of SPY in P20. In addition, for SPY in P40, the GSCA had a significant (*P* < 0.01) positive association with SH and MPH. In P60, the GSCA had a positive interaction with SH, MPH and BPH of SFP. In addition, the association between the GSCA effect and SH of SPY was positively significant (Table S2).

### Combined AMMI analysis of variance of the SPY and the decomposition of GEI Effect

The AMMI analysis for SPY revealed significant differences for both the main effects of genotype and graded levels of applied P over both the year, as well as for their interaction (Table S3). This indicates that there is sufficient variation exists among the genotypes, environments, and their interactions. In terms of variance partitioning, the genotypic variation accounted for 67.98% of the total variance in the AMMI analysis, followed by environmental variation at 25.28%, and genotype-environment interaction (GEI) at 6.74%. A low value of environmental variation indicates expression of the trait was less modified by a given environment. Variation of 74.72% by Genotype and GEI used to detect genotype suitable for narrow adaptation. The GEI effect was further resolved using the AMMI analysis, which provided four interaction principal component analyses (IPCAs). These four IPCAs collectively accounted for 100% of the total GEI effects. Specifically, IPCA1, IPCA2, IPCA3, and IPCA4 explained 70.52%, 25.13%, 2.50%, and 1.45% of the total GEI effects, respectively. All four IPCAs were found to be significant, highlighting their importance in capturing the variation attributed to genotype-environment interaction.

### Effect of graded levels of applied P of both years on the performance of the genotypes

The AMMI analysis provided insights into the mean performance of genotypes for SPY in each graded level of applied P of both year (environment), as well as the environmental principal component analysis (EPCA1) and the top four ranked genotypes in each year phosphorus environment (Table 7). Each graded level of applied P of both year environment recorded the overall mean SPY for genotypes is 17.48 g. Based on the estimated environmental index, graded level of applied P of both year environments was classified as unfavorable or favorable for the cultivation of hybrids and parents. A negative environment index value indicated an unfavorable environment, while *Kharif-*2017 with P40 and *Kharif*-2019 with P60 were classified as favorable. Furthermore, the estimated IPC1 revealed that graded level of applied P of both years responded differently to the stability of hybrids and parents for SPY. Among the graded levels of applied P, *Kharif-*2017 with P20 had the lowest IPC1 estimate, while *Kharif*-2019 with P60 had the highest IPC1 record. This implies that *Kharif*-2019 with applied P60 was the main contributor to the genotypic stability for SPY, while *Kharif*-2017 with applied P20 had the lowest contribution to the genotype-environment interaction.

**Table 7:** AMMI analysis based on Single plant yield means for genotypic performance and environment EPC1 value and the four top ranking genotypes for each applied P (20 kg ha^−1^, 40 kg ha^−1^ and 60 kg ha^−1^) environments.

Envirnoment	Mean	EPC1	Index	Class	I	II	III	IV
*Kharif* 2017 applied P 20 kg ha^−1^	12.29	− 5.2	− 1.75	Unfavorable	H14	H14	H14	H14
*Kharif* 2019 applied P 20 kg ha^−1^	16.31	− 1.17	− 0.83	Unfavorable	H14	H10	H3	H6
*Kharif* 2017 applied P 40 kg ha^−1^	21.65	4.16	2.63	Favorable	H14	H3	H8	H1
*Kharif* 2019 applied P 40 kg ha^−1^	13.83	− 3.65	− 1.81	Unfavorable	H14	H10	H3	H6
*Kharif* 2017 applied P 60 kg ha^−1^	17.84	0.35	− 0.83	Unfavorable	H14	H3	H10	H8
*Kharif* 2019 applied P 60 kg ha^−1^	23	5.51	2.59	Favorable	H14	H3	H8	H1
	GM = 17.48							

### AMMI biplots for GEI

**Fig. 3:**
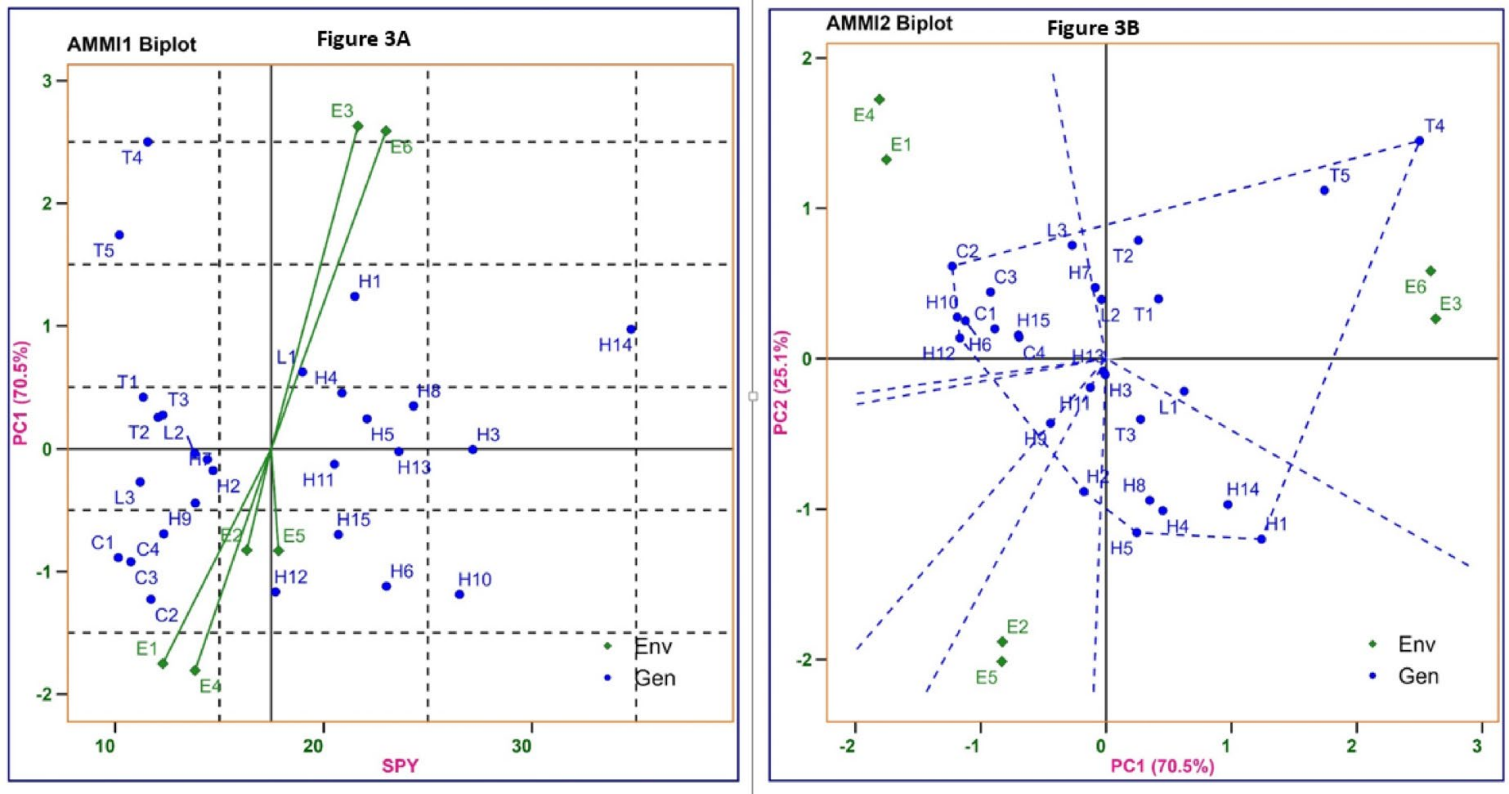
(**a**) AMMI1 Biplot (Mean grain yield vs IPC1) (**b**) AMMI2 Biplot (IPC1 vs IPC2) for SPY of 15 rice hybrids, 8 parental lines, 4 check varieties and six environments of which three graded level of applied P (20,40 and 60 kg ha^−1^) in *Kharif* 2017 and other three in *Kharif* 2019. Where **E1**−  *Kharif*− 2017 applied P20kg ha^−1^, **E2**−  *Kharif*− 2019 applied P20 kg ha^−1^, **E3**−  *Kharif* − 2017 applied P40kg ha^−1^, **E4**−  *Kharif*− 2019 applied P40 kg ha^−1^, **E5**−  *Kharif*− 2017 applied P60 kg ha^−1^, **E6**−  *Kharif*− 2019 applied P60 kg ha^−1^.

The AMMI biplots visually revealed the potential yield of genotypes, their stability levels, and their association with each year graded levels of applied P. In the "grain yield vs IPC1 scores," or AMMI1 plot (Fig. 3a), the association between low applied P tolerant hybrid and graded levels of applied P in both year was depicted. Environments—E2 and E5, with shorter vectors relatively close to the origin, showed weak interaction forces, while environments—E3 and E6, with longer vectors and farther from the origin, exhibited stronger interaction forces. Hybrids H14 (34.76 g), H3 (27.16 g), and H10 (26.52 g) outperformed the overall average single plant yield (SPY) of 17.48 g. The entries closer to the origin of the axis (IPCA1) have a smaller contribution to the interaction than those are far away from the origin. Hybrids H3, H5, and H8 exhibited low positive interaction, and H13, H11, and H15 showed low negative interaction, indicating minimal environmental influence and thus high adaptability across environments. Notably, hybrids H1 and H14 displayed positive interactions with normal phosphorus application (60 kg ha⁻^1^) during *Kharif* 2017 (E3) and *Kharif* 2019 (E6), resulting in higher yields compared to their performance under low P conditions (E1 and E4). On the other hand, hybrids—H2, H7, and H11, along with the line L2, were located closer to the origin, indicating either wide adaptability or insensitivity to environmental conditions with near-average overall mean yielding capacity (17.48 g). The environments with normal phosphorus application (60 kg ha⁻^1^) during *Kharif* 2017 (E3) and *Kharif* 2019 (E6) were positioned in the upper right quadrant of the AMMI biplot, indicating positive interactions and identifying them as favorable environments for higher yield.

In the AMMI2 biplot (Fig. 3b), the first two principal components (IPCA1 and IPCA2) which contributed 70.52% and 25.13% of the total variation, respectively, explained a combined 95.65% of the GEI. Dotted lines related to the vertex genotypes in polygon views demonstrated maximum or minimum SPY with specific environmental adaptability. The degree of interaction with a given environment was determined by a perpendicular projection from the genotype to the environmental vector. Hybrids H13, H3, and H11, located near the origin of the AMMI2 biplot, exhibited greater stability across environments, whereas genotypes such as H1, H2, H5, H12, C2 (Rasi), and T4 (ATR226), positioned farther from the origin, showed higher or lower SPY with unstable performance across varying phosphorus application levels in both years in addition, the Yield Stability Index (YSI), along with AMMI’s stability values and mean SPY, was calculated to classify the genotypes (Table 8). Genotypes or hybrids with high stability values, lower YSI values, and greater productivity were considered superior. Based on YSI, genotypes—H10 (YSI = 8), H14 (YSI = 9), H1 (YSI = 11), H6 (YSI = 6), H12 (YSI = 19), H8 (YSI = 21), H15 (YSI = 21), T4 (YSI = 23), H4 (YSI = 23), and L1 (YSI = 25) exhibited higher stability and SPY, and are identified as top hybrids. Conversely, genotypes such as L2 (IR79156B, YSI = 42), L3 (CRMS32B, YSI = 43), T2 (AR19-42R, YSI = 40), T1 (AYT21, YSI = 39), H7 (YSI = 39), T3 (ATR305, YSI = 39), and C1 (Kasalath, YSI = 37) demonstrated lower stability and poor SPY.

**Table 8:** Mean single plant yield (g), AMMI stability values (ASV) and ranking orders of the parental lines and rice hybrids tested across six environments during *Kharif* 2017 and 2019.

Entry	Mean	PCA I	r Yield (A)	ASI	rASI (B)	YSI (A + B)
L1	18.99	0.62	12	1.21	13	25
L2	13.81	− 0.04	17	0.08	25	42
L3	11.2	− 0.27	24	0.53	19	43
T1	11.35	0.42	23	0.82	16	39
T2	12.06	0.26	20	0.51	20	40
T3	12.29	0.28	19	0.55	18	37
T4	11.56	2.5	22	4.88	1	23
T5	10.2	1.74	26	3.39	2	28
H1 (L1 × T1)	21.5	1.24	8	2.42	3	11
H2 (L1 × T2)	14.69	− 0.18	14	0.35	22	36
H3 (L1 × T3)	27.16	− 0.01	2	0.02	27	29
H4 (L1 × T4)	20.88	0.45	9	0.88	14	23
H5 (L1 × T5)	22.08	0.24	7	0.47	21	28
H6 (L2 × T1)	23.02	− 1.12	6	2.19	7	13
H7 (L2 × T2)	14.42	− 0.09	15	0.18	24	39
H8 (L2 × T3)	24.32	0.35	4	0.68	17	21
H9 (L2 × T4)	13.85	− 0.44	16	0.86	15	31
H10 (L2 × T5)	26.52	− 1.19	3	2.32	5	8
H11 (L3 × T1)	20.52	− 0.13	11	0.25	23	34
H12 (L3 × T2)	17.7	− 1.17	13	2.28	6	19
H13 (L3 × T3)	23.61	− 0.02	5	0.04	26	31
H14 (L3 × T4)	34.76	0.97	1	1.89	8	9
H15 (L3 × T5)	20.71	− 0.7	10	1.37	11	21
C1	10.15	− 0.89	27	1.74	10	37
C2	11.72	− 1.23	21	2.40	4	25
C3	10.75	− 0.92	25	1.79	9	34
C4	12.33	− 0.69	18	1.35	12	30

### GGE biplots

Genotype and genotype plus environment (GGE) biplots developed with the site regression (SREG) model^20,21^ show only genotype main effects along with GEI effects by ignoring random error. It is observed that the first four principal components amount to 99.60% of the total variation explained by genotype and GEI (Table S3). The principal components of above such as PC1 (70.52%) and PC2 (25.13%) jointly have a 95.65% contribution towards total variation by GEI. GGE biplots structured using centering of each year the graded level of applied P of value 2, not scaled and singular value partition for genotype (SVP) is one.

### Which–won where and what

Graphical grouping of each year graded level of applied P was carried out with GEI of high-yielding genotypes (Fig. 3b). GGE biplot constructed using PC1 and PC2 of 95.65% covers three mega environments, each of them having two, such as the first one has E1, E4 and second having E2, E5 and the third has E3, E6. The biplots for H12 are highest yielding in E1 and E4. Genotype H2 in E2, E5 and Tester T4 found high in E3, E6 environments. The sectors with no environment in which H5 and H11 were the vertex hybrids found the lowest yielding at some or across the graded level of applied P. Hybrids situated near to the origin of the polygon are more adapted to low applied P conditions than the vertex hybrids such as H13, H3 and H11.

### Average single plant yield and stability of hybrids

**Fig. 4:**
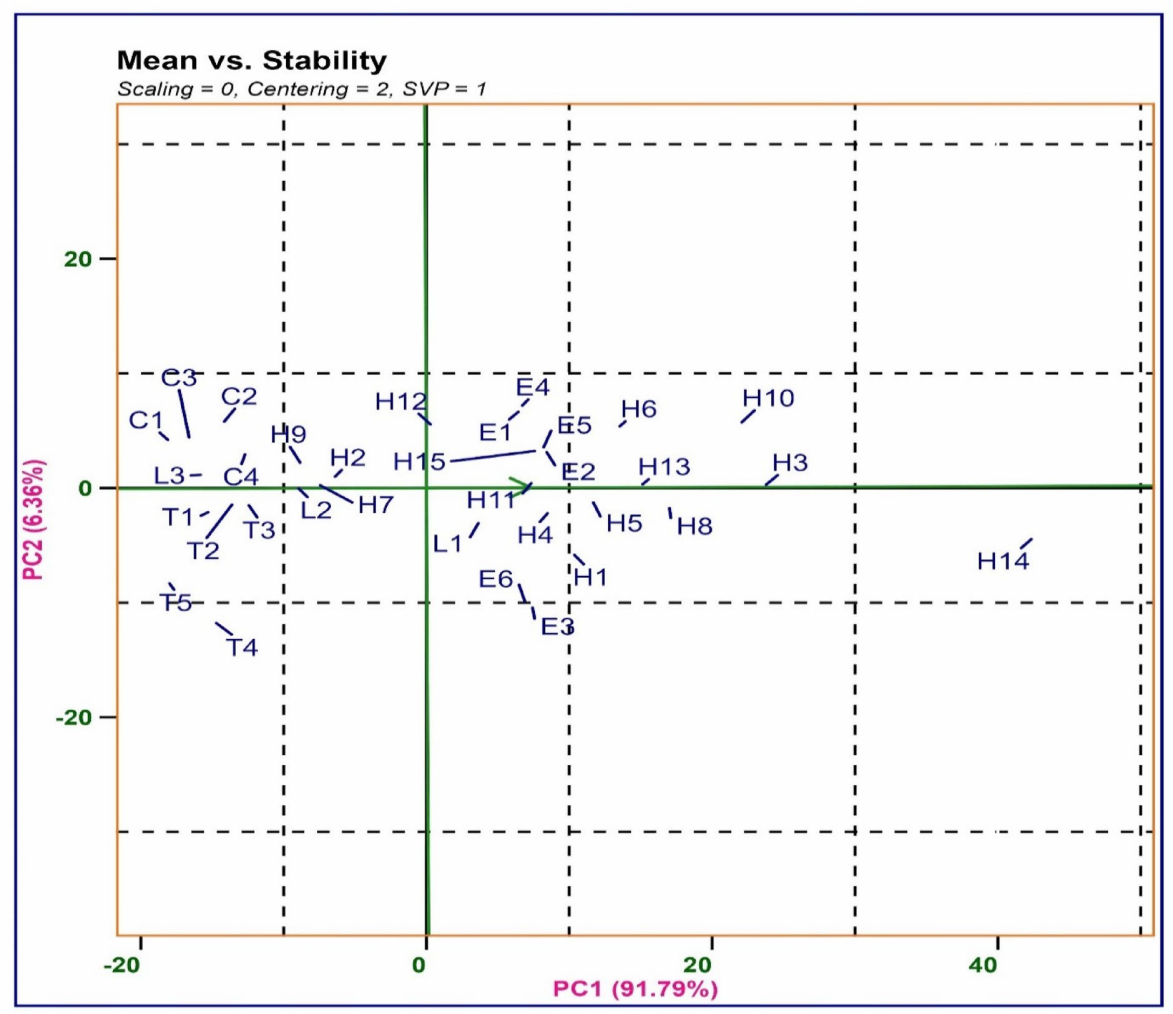
Average environment coordination (AEC) view of the GGE− biplot based on environment− focused scaling for the mean performance and stability of 15 rice hybrids, 8 parental lines, 4 check varieties and six environments with the graded level of applied P (20,40 and 60 kg ha^−1^) during *Kharif* 2017 and 2019. Where **E1**−  *Kharif*− 2017 applied P20kg ha^−1^, **E2**−  *Kharif*− 2019 applied P20 kg ha^−1^, **E3**−  *Kharif* − 2017 applied P40kg ha^−1^, **E4**−  *Kharif*− 2019 applied P40 kgha^−1^, **E5**−  *Kharif*− 2017 applied P60 kg ha^−1^, **E6**−  *Kharif*− 2019 applied P60 kg ha^−1^.

To demonstrate the mean SPY and stability of the hybrids, an AEC (Average Environment Coordination axis) view of the biplot was created (Fig. 4). This plot is particularly suitable for hybrid evaluation because it was generated using genotype-metric preservation (SVP = 1). In the AEC plot, the abscissa (represented by a single-arrowed line) points to a higher mean yield across the environments. Therefore, hybrid H14 exhibited the maximum yield across environments, followed by H3, H10, H8, and H13, while C1 (Kasalath), T5 (TCP795), followed by C3 (IR64) and T1 (AYT21), were noted to be low yielders. The AEC ordinate, represented by a thick solid line perpendicular to the AEC, indicates more variability (poorer stability) in both directions. Thus, entries T4, T5, H6, H10, H12 and C3 exhibited more variability *i.e.,* they were highly unstable, whereas entries H3, H7, H13, H11, L2, and T2 were more stable.

## Discussion

Rice, a staple cereal food crop in the majority of Asian countries, is facing many challenges due to input deficiencies for cultivation, particularly in developing nations. Raising nutrient requirements and increasing costs of fertilizers and other raw materials are major concerns. Phosphorus, is a vital nutrient for crop physiology as an energy carrier nucleic acid material and enhancing crop production. However, Indian soils often exhibit phosphorus deficiency, underscoring the importance of heterosis effects on rice crop productivity under low and moderate levels of applied P.

Presence of non-significant error variance difference across years in each gradient level of applied P for most of the traits except two in which two-way analysis of variance was carried^22^. Consequently, combined analysis was conducted for all traits under study across years, separately for each grade of applied P, utilizing the L × T design for important yield-related traits.

Hybrid performance across years exhibited non-significant differences^22^, with most hybrids demonstrating early flowering compare to parents across applied P levels with late flowering in low P compare to other two graded P level conditions. Genetic variance for the number of productive tillers, spikelet fertility percentage, and hybrid yield, as well as parental and check varieties, decreased from higher to lower levels of applied P^11,7,8,9,24,25^. Few hybrids displayed significant hybrid vigor across graded levels of applied P, outperforming their parents and check varieties in terms of early flowering, plant height, SPY and spikelet fertility percentage under low P. These findings align with previous research, as SPY significantly correlates with NPTP and SFP^25,26^. The remaining hybrids showed reduced numbers of productive tillers and lower spikelet fertility percentages under low phosphorus conditions compared to their parents, consistent with findings under low nitrogen conditions reported by^27^. Hybrids shows higher P uptake and absorption by producing more root length, root biomass and growth related parameters of increased biomass, NPTP, etc. under low P condition and normal condition in maize^24^.

The estimation of variation attributed to combining ability revealed the presence of both types of gene action (additive and non-additive), for the key traits. Across all the traits studied, the variance component estimates of SCA exceeded those of GCA. Moreover, the ratio of mean square components associated with GCA and SCA variance was significantly below one. These findings suggest that non-additive genetic variation was prevalent among the hybrids. Similarly, studies by^28,25^, and ^29^ in soybean under low phosphorus conditions highlight the importance of both additive and non-additive gene actions, with a predominance of non-additive effects for yield-related traits and phosphorus use efficiency (PUE). Comparable findings were reported by^26^ in maize under both low and high P conditions, and by^27^ under nitrogen-limiting and optimal nitrogen conditions. The genotypic variations among the included sets of genotypes, or differences in applied P levels under which the genotypes were evaluated, may contribute to the observed non-additive gene action. Additionally, the inability of statistical models to detect non-additive gene effects could be another contributing factor. Considering the prevalence of non-additive genetic effects for seed yield and its component traits, hybrid development could be strategically employed under low applied P conditions to capitalize on non-additive gene effects, which are often associated with overdominance and epistasis, thereby enhancing heterotic potential (> 21% in rice) rather on pureline selected varieties (predominance of additive gene effects) for low P condition with allelic complementation effects^26^, in addition to presence of commercial system of hybrid production in rice based on Cytoplasmic- genetic male sterility system.

In the majority of cases, testers, or restorer lines, emerged as primary contributors to the variation observed in hybrids because of presence of limited number of cytoplasmic lines of rice in India, exerting significant influence on both inter-hybrid and intra-hybrid variability. Consequently, when selecting parental lines, specific emphasis should be placed on the choice of testers, given their pivotal role in hybrid performance. However, due to the unpredictable nature of Line × Tester interactions, which are beyond the breeder’s control and contingent upon specific patterns of interaction between the lines and testers, the decision-making process becomes more complex. Therefore, cautious consideration is imperative when selecting testers for hybridization. Our study found that lines outperformed testers across graded levels of applied phosphorus for the studied traits, despite contributing less to overall variation. This is likely due to the presence of the *Pup1* QTL in the lines, which enhances phosphorus uptake and utilization efficiency^9^. The GCA effects serve as decisive indicators for identifying parental lines, offering insights into the average performance of a line across a range of hybrid crosses. The per se performance of parental lines often translates into the performance of resulting hybrids^30^. However, in addition to per se performance, the high GCA effects of parental lines are essential for obtaining desirable segregants in breeding programs, especially where additive gene action predominates in the GCA effects, as these effects can be fixed through selfing^13^. ^31^ observed that, after subjecting parental lines to prior testing and selection, the variances for specific combining ability effects assumed greater significance than those for general combining ability effects for hybrid development.

In our study, the GCA effects of lines exhibited variations, with L3 displaying significant positive effects on SPY, enhanced by the NPTP, but negative effects on SFP were observed under low P application levels. Conversely, testers (exception of T1 and T2) demonstrated significant positive GCA effects on SPY. The T3 was found to be the highest GCA for SPY, SFP and reduced tillers, and increased flowering duration as compared to normal applied P. Notably, combining ability was optimal under low P conditions, whereas in medium and normal P application levels yielded contrasting results for yield and its component traits similar with low nitrogen condition by^27,32,33,24^. Line L3 and tester T3 could serve as valuable genetic resources for enhancing rice yield under low P stress by transferring favorable alleles to their progeny. These pure lines used to create superior offspring when crossed with other pureline under low P condition^32^. Negative GCA effects observed between SPY, SFP and NPTP across graded P levels underscored the imperative to improve parental lines for grain yield under low P conditions^35^ observed improvement of parental lines under aerobic condition to enhance performance of hybrids). Positive or negative, a low GCA estimate suggests that the mean of a parent in a cross with the other does not deviate much from the overall mean of the crosses. The parental mean is either higher or lower to the overall mean; however, according to a high GCA estimate the parental mean is superior or inferior to the general mean. This gives information about the concentration of predominant genes with additive effects^36^.

Rice genotypes harboring dominant genes with negative effects tend to exhibit early maturation, a desirable trait with regard to DFF. Notably, lines such as L2 (IR79156B) and tester T1 (AYT21) demonstrated early DFF across graded P levels, consistent with previous findings by^37,38^, and they identified L2 as the best general combiner for earliness under normal P condition. The negative GCA results of these lines showed potential utility in breeding programs aimed at developing early maturing lines for N Uptake and N use efficiency in low N soil^33^.

In contrast to GCA effects, SCA is typically associated with non-additive gene action, including dominance, overdominance, and epistasis^13,34^. Analysis of hybrids revealed that most of the combination didn’t produced substantial positive SCA values for all evaluated characters, only five hybrids in low P and four hybrids in both medium and normal P condition showed positive SCA value^32,39^. However, the hybrid H14 (CRMS32A × ATR226) exhibited significant positive SCA for SPY, SFP and NPTP across graded level of applied P where both parents displayed positive GCA effects for yield due to additive × additive gene action but for other traits parents varies with positive and negative GCA effects, moreover presence of K46-2 and K59 gene of *Pup1* QTL of Rice in the parents^9^. H12 (CRMS32A × AR19-42R) found higher significant positive SCA for SPY, SFP across graded P level and for NPTP showed positive in low, normal applied P and negative in medium applied P but late flowering where parents have positive (line) and negative GCA (tester) for SPY with presence of K46-2 and K46-1, K46-2, K59 of *Pup1* QTL Rice, respectively^9^. Hybrids exhibited mixed performance, with higher specific combining ability (SCA) effects under low phosphorus compared to normal phosphorus, and the reverse also observed in some cases. Similar responses under low and normal nitrogen conditions were reported by^32^. Our study highlights promising specific cross combinations from parents with varying general combining abilities, non-additive effects of hybrids due to allelic complementation and difference of gene frequencies in parents^26,32,39^. The evaluation of fifteen hybrids was insufficient to identify a majority of hybrid individuals exhibiting dominant phenotypes under low phosphorus conditions. Meanwhile GCA and SCA in the study found to be inconsistent with graded level of applied P. therefore identifying best GCA parents and SCA hybrids separately in low and normal P helps genetic gain of yield, better P uptake and P use efficient^33^.

The sum of GCAs of parental lines of hybrids represents the GSCA (General Sum of Combining Ability) value^40^. GSCA had a positive association with SCA and heterosis traits for SPY in across the graded levels of applied P. These results are similar to^40,35^ and contrast with^15^.

Heterosis studies revealed various hybrids exhibiting MPH, BPH and SH for all the studied traits, suggesting the potential use of heterosis to increase yield under graded P levels. Significant MPH observed in many crosses for several traits indicates the presence of additive gene action, consistent with findings reported by^41^. Furthermore, our study has found a higher level of heterosis in low P in comparison to normal P condition as reported by^27^ in maize and^42^ in sorghum hybrids. Better parent heterosis has yield advantage with a highest in low p condition due to dominance effect over best performance line in the study in compared to normal P condition, indirectly reflects the potentiality of hybrid under low P condition^41^.

Based on the per se performance for yield, two potential hybrids were identified. H14 (CRMS32A × ATR226) exhibited markedly significant yield potential with significantly higher MPH, BPH and SH over the standard check (Kasalath). Another promising hybrid, H10 (IR79156A × TCP795), recorded significantly higher SFP with higher significant SH over Kasalath. The higher heterosis observed in these hybrids is positively correlated with higher SFP and NPTP^41^ reported higher heterosis related with remobilization of N from vegetative stage in maize).

The primary objective of stress breeding strategies is to identify stable genotypes with high-performance key traits. In addition to focusing on yield or other individual traits, stability measures such as parametric and non-parametric stability measures, the AMMI stability model, and GGE biplots elucidate genotype stability and performance. The impacts of environments, genotypes, and genotype-environment interaction (GEI) varied significantly, underscoring substantial differences between target environments. A crossover interaction influencing the response and ranking of hybrids across graded P levels was observed when significant GEI was combined with ANOVA, IPC from AMMI, and GGE biplot findings^43^. In AMMI analysis, the first two IPCs accounted for approximately 95.65% of the variance from GEI, while in GGE biplot analysis, IPC1 and IPC2 accounted for 70.52% and 25.13%, respectively. The vectors of low P and moderate P level conditions exhibited a positive correlation, as illustrated by the acute angle between them. Stable genotypes were ranked based on lower absolute IPC1 scores^44^. Low P condition in 2017 and 2019 appears in low yielding environment indicates the effectiveness of imposed stress, And normal P condition of 2017, 2019 found high yielding environment^45^. Selection parameters such as yield stability index (YSI), in conjunction with a higher yield, were utilized to identify stable rice hybrids across graded levels of P application^46^. The selection parameters, including high mean grain yield and stability based on GGE biplot analysis, align with findings in rice (^44^ for drought stress) and maize (^45^ for Nitrogen stress, ^46^ for moisture stress). Stable and high-performing Nagina-22 rice mutant lines for three soil conditions—normal irrigated, water-limiting, and low P content were identified over twelve seasons using AMMI and GGE biplot analysis^47^.

The most stable genotypes in our study were hybrids—H3 (APMS6A × ATR305), H13 (CRMS32A × ATR305), and H11 (CRMS32A × AYT21), showing higher heritability of yield traits and wider adaptation under graded levels of applied P and can perform uniformly in any of the environment without the influence of the environmental effects on yield. Another hybrid, H14 (CRMS32A × ATR226), found highest heterotic potential exclusively in low P with low stable across graded P levels.

### The mechanisms for the efficient use of P in rice hybrids

Low P condition of soil makes differences in genetic potential of studied genotypes. Hybrids observed better root architecture with increase root length and volume, production of organic acids helps in uptake of P in compare to parental lines. Moreover, better response of line GCA for yield traits due to harboring of regions of Pup1 QTL and transmitting ability of lines to progeny compare to testers. Hybrids respond well over parents with better vegetative biomass, tall plant, more productive tillers, increased chlorophyll, good leaf expansion which are observed with higher SCA effect in low P due to dominance gene action ^24^. Better parent heterosis and standard heterosis was higher in low P condition which indicates P homeostasis from old or senescence parts of leaf and stem towards more grain filling in hybrids in compare to parents. Target breeding of hybrid development under low P for increased enhanced P uptake and P use efficiency will increase yield level.

### Better breeding strategy for P use efficiency

Presence of low P soil and immobilization nature of applied P in rice field causes in variable response among developed hybrids, its parental lines and good estimation of heterosis in this environment under the study. Similar greater discrimination among genotypes for the studied traits was reported ^7,8,9,24^. Therefore, identification and selection of hybrids under graded level of applied P may be significant. Furthermore, estimation of mean square, general and specific combining ability showed most of genetic variation in studies is due to combining abilities of parental lines rather than graded level of applied P. predominance of non-additive gene action across graded level of applied P for studied traits reveals same strategy of crop improvement followed especially heterosis exploitation leads yield advantage.

## Conclusion

Exploiting stress yield heterosis in rice presents a viable strategy to overcome dependency on phosphatic fertilizers and thereby lower production costs. Combining ability revealed predominance of SCA in hybrids, with both positive and negative GCA effects observed among parents, Heterosis estimates under low phosphorus (P) conditions were higher compared to normal P conditions. Among the hybrids, H14 exhibited the highest heterosis across all levels of applied P**,** followed by H10 under low and medium P**,** and H13 under normal P conditions**.** Further, AMMI analysis identified H3 and H13 as stable performers across varying P levels. These findings underscore the importance of developing hybrids specifically adapted to low P environments to enhance phosphorus use efficiency. To validate their performance, the developed hybrids should undergo multi-location trials to assess their adaptability across diverse agro-climatic zones. By deploying these stable and efficient hybrids, farmers can potentially reduce the frequency of phosphatic fertilizer application for 2–3 cropping seasons**,** thus reducing the overall cost of cultivation without compromising grain yield**.** This strategic approach aligns with sustainable agricultural practices and resource-efficient crop production.

## Materials and methods

### Plant materials and field performance evaluation

The experimental materials were chosen based on genotyping for *Pup-1* markers, their phenotypically acceptable performance across graded levels of applied phosphorus, and the data from test cross nurseries assessing fertility restoration. A total of 15 test crosses were developed by following the Line × Tester mating design ^16^ using three CMS lines and five restorer lines. The hybrids (crosses), testers (T—male parent’s/Restorer lines), lines (L—female parent’s/CMS lines) and varietal checks, Kasalath (C1), Rasi (C2), IR64 (C3) and DRR Dhan 42 (C4) were evaluated at Dr. Krishnamurti P Screening facility of Rajendranagar Farm of ICAR- Indian Institute of Rice Research (ICAR-IIRR), Hyderabad, India (17.19 N’ Latitude and 78.23E’ Longitude, 542 m above mean sea level) during wet seasons (*Kharif*)—2017 and 2019. Corresponding maintainer (B) lines were used to denote the Cytoplasmic male sterile (CMS) lines. The list of hybrid crosses, parental lines and checks are indicated in Table 9.

**Table 9:** Lines (L), Testers (T) and Hybrids (H) were used in the L × T mating design study in graded levels of applied P (20 kg ha^−1^, 40 kg ha^−1^ and 60 kg ha^−1^) environments.

Lines	
L1	APMS6B
L2	IR79156B
L3	CRMS32B
**Testers**	
T1	AYT− 21
T2	AR19− 42R
T3	ATR− 305
T4	ATR− 226
T5	TCP− 795
**Hybrids/Crosses**	
H1	APMS6A × AYT21
H2	APMS6A × AR19− 42R
H3	APMS6A × ATR305
H4	APMS6A × ATR226
H5	APMS6A × TCP795
H6	IR79156A × AYT21
H7	IR79156A × AR19− 42R
H8	IR79156A × ATR305
H9	IR79156A × ATR226
H10	IR79156A × TCP795
H11	CRMS32A × AYT21
H12	CRMS32A × AR19− 42R
H13	CRMS32A × ATR305
H14	CRMS32A × ATR226
H15	CRMS32A × TCP795
**Checks**	
C1	Kasalath
C2	Rasi
C3	IR64
C4	DRR Dhan 42

The experiment was executed at the graded levels of P plot 20 kg ha−1, 40 kg ha−1 and 60 kg ha−1^20,40,1^ of Dr. Krishnamurti P Screening facility at Rajendranagar farm of ICAR-IIRR. The soil properties are mentioned in Table 10.

**Table 10:** Soil Properties of graded level of Applied P in different Years.

Soil properties	*Kharif* 2017	*Kharif* 2019
Soil Type	Clayey Vertisol (Typic Pellustert)	Clayey Vertisol (Typic Pellustert)
pH	7.94	7.81
Organic carbon (%)	0.58	0.55
Electrical conductivity (dS/m)	0.80	0.72
Available Nitrogen (kg/ha)	235	221
Available Potassium (kg/ha)	450	428
Available P in P20 (kg/ha)	6.1	6.2
Available P in P40 (kg/ha)	18.9	19.2
Available P in P60 (kg/ha)	27.4	28.0

Twenty-four days old seedlings were used for main field transplantation at the rate of one seedling per hill. Recommended cultural operations were taken up to ensure uniform and healthy crop stand as per package of practices. The CMS plant spikelets that were anticipated to flower next day were chosen and the top one third was clipped to improve the good seed set. Clipped panicles were covered with butter paper covers to avoid cross pollination. The next morning (about 8:30 am) panicles from the male parents were collected and kept in a pollination room which had been maintained in hot and humid conditions. The pollen grains were dusted carefully over clipped spikelets of CMS plants. Immediately pollinated panicles were covered by butter paper along with a label of the date of pollination and crossed parents. After 30 days of hybridization, covered panicles were detached from plants and each cross seeds were separated and shade dried.

All 15 hybrids, their parents and four varietal checks were sown in the nursery seed bed (Table 9). Twenty-one-day-old seedlings were transplanted into graded phosphorus (P) plots measuring 3 × 4.5 m^2^, corresponding to P application levels of 20, 40, and 60 kg ha⁻^1^. Phosphorus was applied as a basal dose using single super phosphate at 169 g, 338 g, and 506 g per plot for 20, 40, and 60 kg P levels, respectively, and broadcasting done uniformly before transplanting. Nitrogen and potassium were applied uniformly across all treatments using urea (352 g per plot, split into three doses) and muriate of potash (90 g per plot as a basal dose), respectively. The experiment was carried out in two replicates of randomized complete block design (RCBD) with spacing of a 20 × 10 cm. the mean yield of three plants selected randomly each from replication comprising 15 hybrids with eight parental varieties and four standard check varieties of the four rows of 2 m each (leaving border rows) were used for the observations viz., Days to 50% flowering (DFF) was recorded as the number of days required for 50% of the plants in the rows to flower from the date of sowing. The Number of productive tillers per plant (NPTP) was counted as the average of the number of tillers that had filled grain in their panicles out of the total tillers of three randomly selected plants. Spikelet fertility percentage (SFP) was calculated as the average of the ratio of filled grains to the total number of filled and chaffy spikelets from the panicles of three randomly selected plants. Single plant yield (SPY), expressed in grams, is determined by averaging the grain yield of three randomly selected plants with a seed moisture content of 12–13%.

### Statistical analysis

#### Analysis of variance

A separate ANOVA was conducted for each year using data from experimental materials of graded P plots. To assess the homogeneity of error variance, data from two years of replicated experiments conducted at graded levels of applied phosphorus were subjected to Bartlett’s test (^48^). The non-significance in Bartlett’s test suggests that the error variance in the year-wise analyses is generally homogeneous, with exceptions observed for the traits of "days to 50% flowering" at applied phosphorus levels of 40 and 60 kgha^−1^, as well as "single plant yield" at an applied phosphorus level of 40 kgha^−1^. Significant interactions between years and genotypes were detected through a two-way analysis of variance ^22^.

#### Combining ability

Analysis of variance for combining ability involved computing mean values across graded levels of applied phosphorus ^16^ to assess differences among genotypes, including crosses and parents ^49^. The sum of squares for hybrids was partitioned into variations arising from lines, testers, and Line × Tester interactions. Mean squares for year × line and year × tester were compared against mean squares for year × line × tester, and the latter against pooled error. Standard errors for the GCA effects of females (lines) and males (testers) and the SCA effects were calculated using the method described by ^50^. Two-tailed t-tests were used to test the significance of the GCA and SCA effects.

Estimates of general combining ability (σ^2^ GCA) and specific combining ability (σ^2^ SCA) were obtained ^51^. Ratios of mean square components associated with variance of GCA and SCA effects were computed as suggested by ^52^ to estimate the relative importance of GCA in explaining performance. The closer the ratio is to unity, the greater the predictability of progeny performance based on GCA effects alone.

The concept of GSCA (General Specific Combining Ability) was introduced by ^15^ in heterosis breeding. It represents the sum of General combining abilities (GCA) for the two parents of a cross combination. GSCA offers an alternative approach to assessing combining ability, leveraging GCA values. This method provides certain advantages, potentially offering insights into the genetic potential of hybrid combinations. The contribution of lines, testers and their interaction towards the total variability percentage for each trait was calculated by ^51^.

#### Heterosis

Replicated year mean data on various quantitative traits of parents and hybrids were used to estimate the percent increase or decrease of F_1_ hybrid over mid-parent (MPH), better parent/ heterobeltiosis (BPH) and standard heterosis over commercial variety (SH) as suggested by ^53^. Further to the find significance of the difference of F_1_ from MP, BP and commercial variety was carried by T- test statistics given by ^54^.

### Stability analysis

#### GEI analysis

For identifying stable genotypes across the graded applied phosphorus condition having six environments carried with the AMMI method ^55^. Analysis of variance and principal component analysis (PCA) included in the AMMI method to make a single approach to analysis ^56,57^. General ANOVA reveals the additive main effects of genotypes and test environments and followed by PCA which accounts for the non-additive portion, genotype by environment interaction (GEI). AMMI method identifies the statistically significant stable genotypes in each environment by ^58^. AMMI biplots graph drawn using the main effect of means versus the first principal component axis (PCA1) and between the first two principal component axes (PCA1 *vs* PCA2). ^59^ suggested the AMMI equation for T genotypes and S environments as;

$${\text{Yij}} = \mu + {\text{gi}} + {\text{ej}} + \Sigma _{{{\text{k}} = 1}}^{{\text{n}}} \lambda _{{\text{k}}} \alpha _{{{\text{ik}}}} \gamma _{{{\text{jk}}}} + \theta _{{\text{i}}}$$

where, Yij is the mean yield of the genotype i (i = 1,2,..,T) in the jth environment (j = 1,2,..,S); μ is the general mean, g_i_ is the ith genotypic effect; e_j_ is the jth location effect; λ_k_ is the eigenvalue of the PCA axis k; γ_ik_ is the ith genotype eigenvector value for IPC axis k, δ_jk_ is the jth environment eigenvector value for IPC axis k, and θ_ij_ is the error term.

The eigen value (EV) stability parameter of AMMI ^60^ was calculated according to the expression:

$$EV = \sum\limits_{n = 1}^{N} {y_{in}^{2} } /n$$

where, γ_in_ is the genotype eigenvector for axis n, and N is the number of IPCs that were retained in the AMMI procedure via different F-test. The sum of IPC scores (SIPC) parameter is expressed as ^61^:

$$SIPC = \sum\limits_{n = 1}^{N} {y_{n}^{0.5} } \cdot y_{in}$$

where, *n* is the eigen value of the IPC analysis axis *n*. In this equation, *N* = 1 for *SIPC*1; and *SIPCF*, *N* was the number of IPCs that were retained in the AMMI model.

ASV (AMMI stability value) was calculated as described by ^62^ as follows

$$\text{ASV}=\sqrt{\left(\frac{SSIPC1}{SSIPC2}\right.{\left(IPC1\right)}^{2}+{\left(IPC1\right)}^{2}}$$

where *SSIPC*1/*SSIPC*2 is the weight given to the *IPC*1 value by dividing the *IPC*1 sum of square by the *IPC*2 sum of square. The larger IPC score, either negative or positive, the more specifically adapted a genotype to certain environments. Smaller ASV scores indicate a more stable genotype across environments.

### Construction of GGE biplot

Single plant yield data from graded applied P were subjected to the construction of GGE biplot according to the model ^63,64^ based on singular value decomposition (SVD) of the first two principal components by ignoring random error is Y_ij_ = μ + β_j_ + α_i_ + θ_ij_ + *ε*_ij_ where, Y_ij_ is the value of the mixed effect of the grand mean (μ) modified by the genotype main effect (α_i_), the environment main effect (β_j_), and the genotype by environment interaction due to genotype i and environment j (θ_ij_), plus any random error (ε_ij_) GGE (Genotype Main Effect plus Genotype-Environment Interaction) biplots were constructed based on site regression analysis, SREG ^65^, GGE biplot tools were used to identify highly adaptable rice hybrids with maximum mean yield by ‘Mean *vs* Stability’ ^66^. The average-environment coordinate (AEC) view of the GGE biplot revealed the high mean yielders and stable genotypes through AEC abscissa and AEC ordinate, respectively. The ‘which-won-where’ pattern, an intrinsic property of the GGE biplot exhibited by the inner-product property of the biplot of genotype by environment data set was also visually presented. Genotypes with PCA1 scores more than zero were considered as high yielders with maximum adaptability whereas less than zero were identified as lower yielding hybrids and non-adaptable ^67^.

## Supplementary Information

Below is the link to the electronic supplementary material.

Supplementary Material 1

## Data Availability

Correspondence and request for data availability should be addressed to N.M and P.S
