# Supplementary material for: Estimation of heterosis and combining ability under low soil phosphorus condition in rice (Oryza sativa L.)
Source: Sci Rep. 2025 Oct 21;15:36623. doi: 10.1038/s41598-025-20344-8 (PMC12541075; doi:10.1038/s41598-025-20344-8)

**Table S1.** Pearson’s correlation between phenotype, heterosis and combining ability effects for studied traits derived from line × tester mating design in graded levels of applied P (P20 kg ha^-1^, P40 kg ha^-1^and P60 kg ha^-1^) environments.

|  | **P20** | | | | **P40** | | | | **P60** | | | |
| --- | --- | --- | --- | --- | --- | --- | --- | --- | --- | --- | --- | --- |
|  | **DFF** | **NPTP** | **SFP** | **SPY** | **DFF** | **NPTP** | **SFP** | **SPY** | **DFF** | **NPTP** | **SFP** | **SPY** |
| **GSCA versus** |  |  |  |  |  |  |  |  |  |  |  |  |
| GCA | -0.454 | -0.054 | 0.058 | 0.195 | -0.372 | 0.075 | -0.05 | -0.163 | -0.233 | 0.012 | -0.032 | -0.119 |
| SCA | 0.108 | 0.157 | -0.304 | -0.836* | 0.024 | -0.401 | -0.215 | -0.698 | 0.624 | -0.176 | -0.162 | -0.358 |
| SH | 0.465 | 0.882* | 0.756* | 0.819* | 0.405 | 0.737* | 0.659 | 0.947* | 0.575 | 0.587 | 0.828* | 0.935* |
| MPH | 0.437 | 0.781* | 0.543 | 0.858* | 0.327 | 0.638 | 0.627 | 0.792* | 0.415 | 0.383 | 0.788* | 0.636 |
| BPH | 0.491 | 0.657 | 0.673 | 0.759* | 0.448 | 0.577 | 0.661 | 0.512 | 0.453 | 0.414 | 0.827* | 0.515 |
| PHENOTYPE | 0.596 | 0.22 | 0.202 | -0.292 | 0.547 | 0.604 | 0.004 | 0.053 | 0.353 | 0.864* | -0.418 | -0.431 |
| **GCA versus** |  |  |  |  |  |  |  |  |  |  |  |  |
| SCA | -0.031 | -0.596 | 0.635 | 0.021 | 0.575 | 0.239 | 0.694 | 0.173 | 0.556 | 0.066 | -0.093 | -0.489 |
| SH | -0.37 | -0.428 | 0.45 | 0.501 | 0.365 | 0.18 | 0.522 | -0.028 | 0.371 | 0.077 | 0.268 | 0.006 |
| MPH | -0.551 | -0.087 | 0.553 | 0.488 | 0.32 | 0.471 | 0.485 | 0.013 | 0.271 | 0.39 | 0.173 | 0.304 |
| BPH | -0.585 | 0.096 | 0.41 | 0.653 | 0.216 | 0.599 | 0.564 | 0.404 | 0.211 | 0.596 | 0.266 | 0.375 |
| PHENOTYPE | -0.515 | -0.042 | 0.287 | -0.451 | 0.02 | 0.232 | 0.121 | -0.104 | 0.33 | 0.205 | 0.153 | 0.16 |
| **SCA versus** |  |  |  |  |  |  |  |  |  |  |  |  |
| SH | 0.178 | 0.346 | 0.194 | -0.646 | 0.278 | -0.167 | 0.437 | -0.652 | 0.740* | 0.503 | 0.101 | -0.26 |
| MPH | -0.019 | -0.078 | 0.463 | -0.589 | 0.033 | -0.012 | 0.399 | -0.398 | 0.476 | 0.589 | 0.172 | -0.151 |
| BPH | -0.057 | -0.322 | 0.297 | -0.413 | 0.038 | 0.055 | 0.364 | -0.117 | 0.473 | 0.546 | 0.105 | -0.107 |
| PHENOTYPE | 0.434 | -0.402 | 0.26 | -0.162 | 0.17 | 0.024 | -0.115 | -0.045 | 0.321 | -0.059 | -0.035 | 0.693 |

**Table S2**. Estimation of relative GSCA (General Sum of Combining Ability) effects of crosses (Hybrids) for DFF, NPTP, SFP and SPY in Line × Tester (Including parents) mating design in graded levels of applied P (20,40 and 60 kg ha^-1^) conditions*.* Where DFF - Days to 50% flowering; NPTP- Number of productive tillers per plant; SFP- Spikelet fertility percentage; SPY - Single plant yield (g); L1 to L3 - Lines; T1 to T5 - Testers; H1 to H15 - Hybrids. P20 - P 20 kg ha^-1^; P40 - P 40 kg ha^-1^; P60 - P 60 kg ha^-1^

| **Entries** | | **DFF** | | | **NPTP** | | | **SFP** | | | **SPY** | | |
| --- | --- | --- | --- | --- | --- | --- | --- | --- | --- | --- | --- | --- | --- |
|  |  | **P20** | **P40** | **P60** | **P20** | **P40** | **P60** | **P20** | **P40** | **P60** | **P20** | **P40** | **P60** |
| **Hybrids** | H1 (L1 × T1) | -7.17 | -1.07 | -5.66 | -1.03 | -1.08 | -0.67 | 1.62 | 2.71 | 0.32 | -2.29 | -0.01 | 0.84 |
|  | H2 (L1 × T2) | -1.08 | 1.43 | 2.34 | -0.59 | -1.04 | -0.62 | -10.58 | -8.61 | -8.52 | -7.08 | -6.25 | -6.36 |
|  | H3 (L1 × T3) | 1.25 | 1.52 | -1.41 | -1.39 | -1.25 | -0.89 | 3.72 | 4.03 | 0.6 | 0.9 | 3.22 | 4.47 |
|  | H4 (L1 × T4) | 5.25 | 3.94 | 4.5 | -0.96 | -1.07 | -0.12 | -3.68 | -0.7 | 0.05 | -2 | 1.99 | 3 |
|  | H5 (L1 × T5) | -2.5 | -0.81 | -1.41 | 0.42 | 0.18 | -0.15 | -1.57 | 2.07 | -2.85 | 0.32 | 1.6 | 0.9 |
|  | H6 (L2 × T1) | -7.92 | -2.53 | -7.61 | 0 | 0.55 | 0.16 | 6.9 | 5.24 | 4.96 | -0.4 | -1.7 | -1.88 |
|  | H7 (L2 × T2) | -1.83 | -0.03 | 0.39 | 0.44 | 0.59 | 0.21 | -5.3 | -6.08 | -3.88 | -5.19 | -7.94 | -9.08 |
|  | H8 (L2 × T3) | 0.5 | 0.06 | -3.36 | -0.36 | 0.38 | -0.06 | 9 | 6.56 | 5.24 | 2.79 | 1.53 | 1.75 |
|  | H9 (L2 × T4) | 4.5 | 2.48 | 2.55 | 0.07 | 0.56 | 0.71 | 1.6 | 1.83 | 4.69 | -0.11 | 0.3 | 0.28 |
|  | H10 (L2 × T5) | -3.25 | -2.27 | -3.36 | 1.45 | 1.81 | 0.68 | 3.71 | 4.6 | 1.79 | 2.21 | -0.09 | -1.82 |
|  | H11 (L3 × T1) | -3.87 | 1.38 | -2.71 | 0.07 | -0.16 | -0.02 | 2.64 | 1.38 | 1.92 | 1.91 | 1.36 | 1.86 |
|  | H12 (L3 × T2) | 2.22 | 3.88 | 5.29 | 0.51 | -0.12 | 0.03 | -9.56 | -9.94 | -6.92 | -2.88 | -4.88 | -5.34 |
|  | H13 (L3 × T3) | 4.55 | 3.97 | 1.54 | -0.29 | -0.33 | -0.24 | 4.74 | 2.7 | 2.2 | 5.1 | 4.59 | 5.49 |
|  | H14 (L3 × T4) | 8.55 | 6.39 | 7.45 | 0.14 | -0.15 | 0.53 | -2.66 | -2.03 | 1.65 | 2.2 | 3.36 | 4.02 |
|  | H15 (L3 × T5) | 0.8 | 1.64 | 1.54 | 1.52 | 1.1 | 0.5 | -0.55 | 0.74 | -1.25 | 4.52 | 2.97 | 1.92 |

**Table S3.** AMMI analysis of variance for grain yield among parental lines, hybrids and checks across six test environments derived out of graded levels of applied P during *Kharif* 2017 and *Kharif* 2019.

| **Source of variation** | **Df** | **Sum Sq** | **Mean Sq** | **Proportion** | **Accumulated** |
| --- | --- | --- | --- | --- | --- |
| **Rep. (Env)** | 161 | 9566.63 | 59.42 |  |  |
| **GEN** | 26 | 6503.51 | 250.14 | 67.98 |  |
| **ENV** | 5 | 2418.55 | 483.71 | 25.28 |  |
| **GEN:ENV** | 130 | 644.58 | 4.96 | 6.74 |  |
| **IPC1** | 30 | 454.55 | 15.15 | 70.52% | 70.52% |
| **IPC2** | 28 | 161.98 | 5.79 | 25.13% | 95.65% |
| **IPC3** | 26 | 16.12 | 0.62 | 2.50% | 98.15% |
| **IPC4** | 24 | 9.33 | 0.39 | 1.45% | 99.60% |
| **Residuals** | 22 | 2.6 | 0.12 | 0.40% | 100.00% |
| **Totals** | 130 | 190.03 | 1.9 |  |  |

**Figure S1.** Principal component analysis (PCA) biplot diagrams for parents representing General Combining Ability (GCA) and mean values of traits in P 20 kg ha^-1^ . 1 to 3 indicates the Lines L1 to L3; 4 to 8 indicates the Testers T1 to T5.


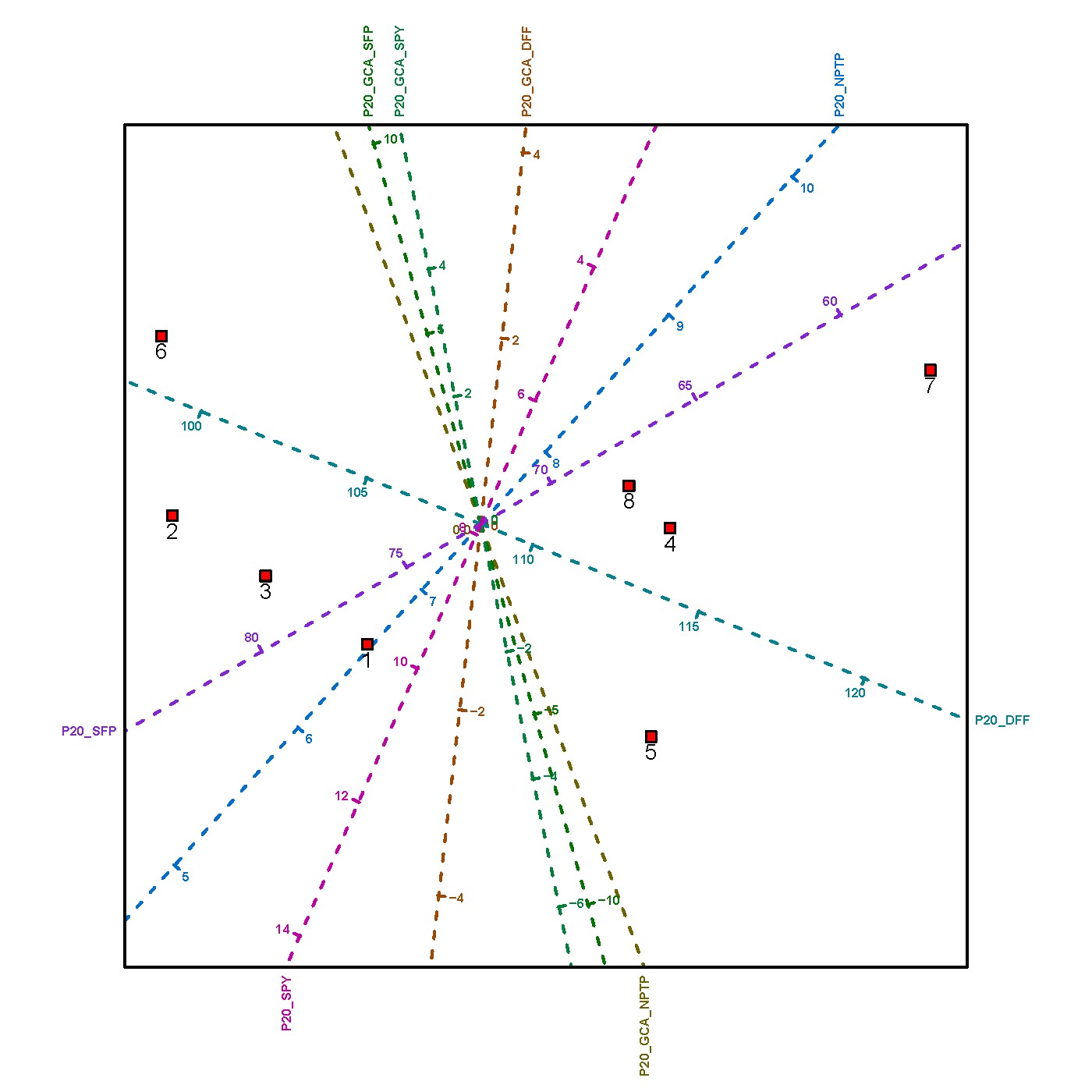


**Figure S2.** Principal component analysis (PCA) biplot diagrams for parents representing General Combining Ability (GCA) and mean values of traits in P 40 kg ha^-1^. 1 to 3 indicates the Lines L1 to L3; 4 to 8 indicates the Testers T1 to T5.


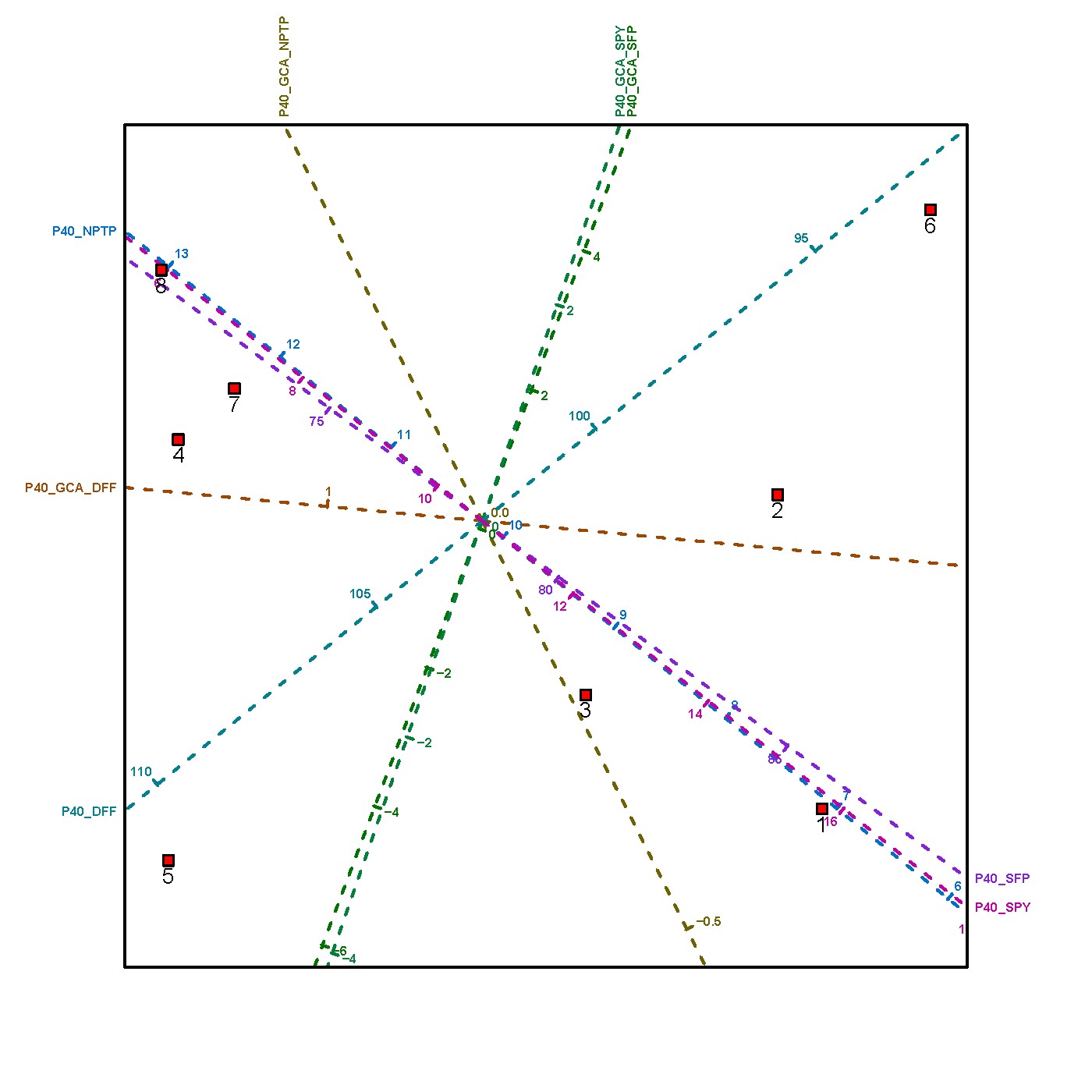


**Figure S3.** Principal component analysis (PCA) biplot diagrams for parents representing General Combining Ability (GCA) and mean values of traits in P 60 kg ha^-1^. 1 to 3 indicates the Lines L1 to L3; 4 to 8 indicates the Testers T1 to T5.


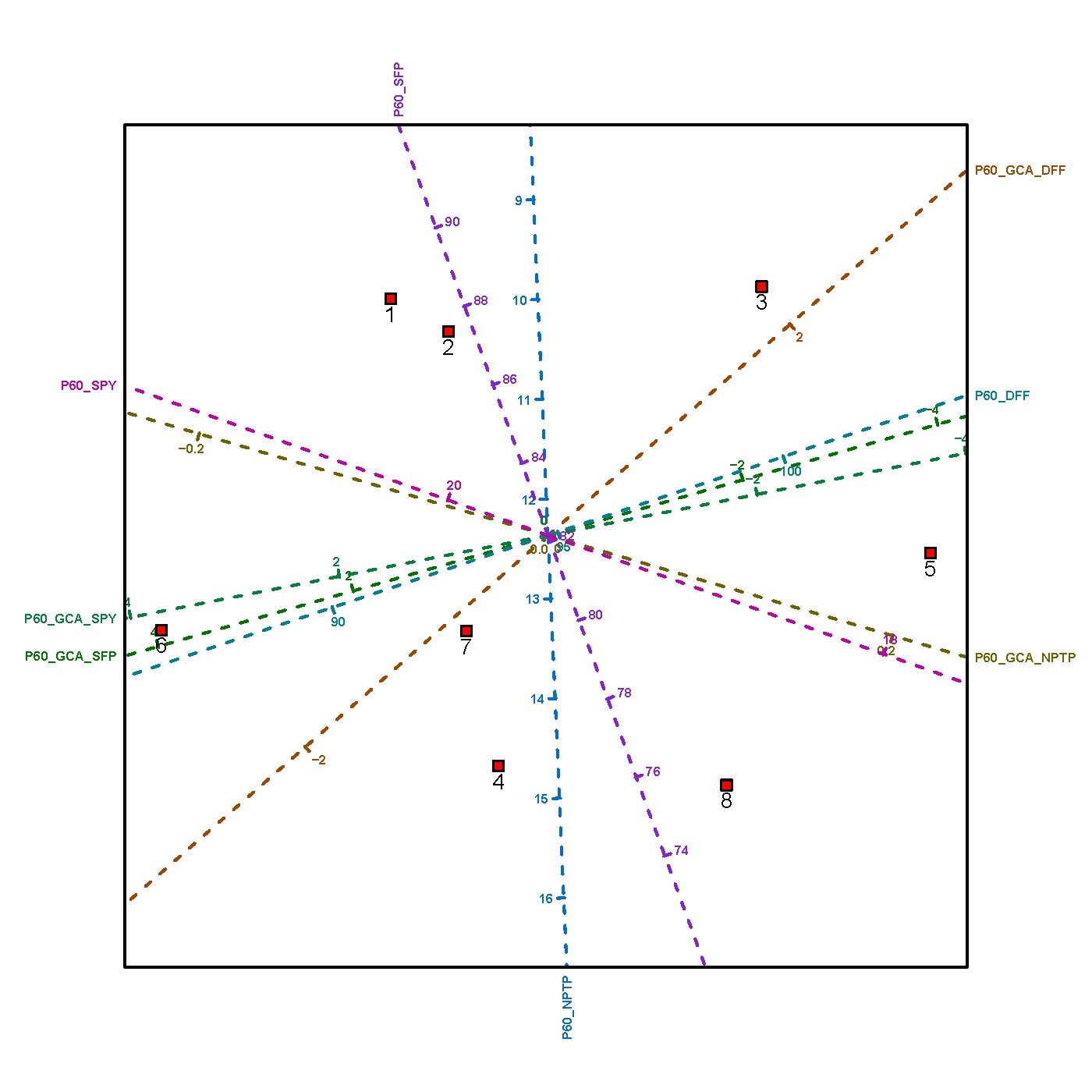


**Figure S4.** Principal component analysis (PCA) biplot diagrams for crosses representing Specific Combining Ability (SCA) and mean values of traits in P 20 kg ha^-1^. 1 to 15 indicates the hybrids H1 to H15.


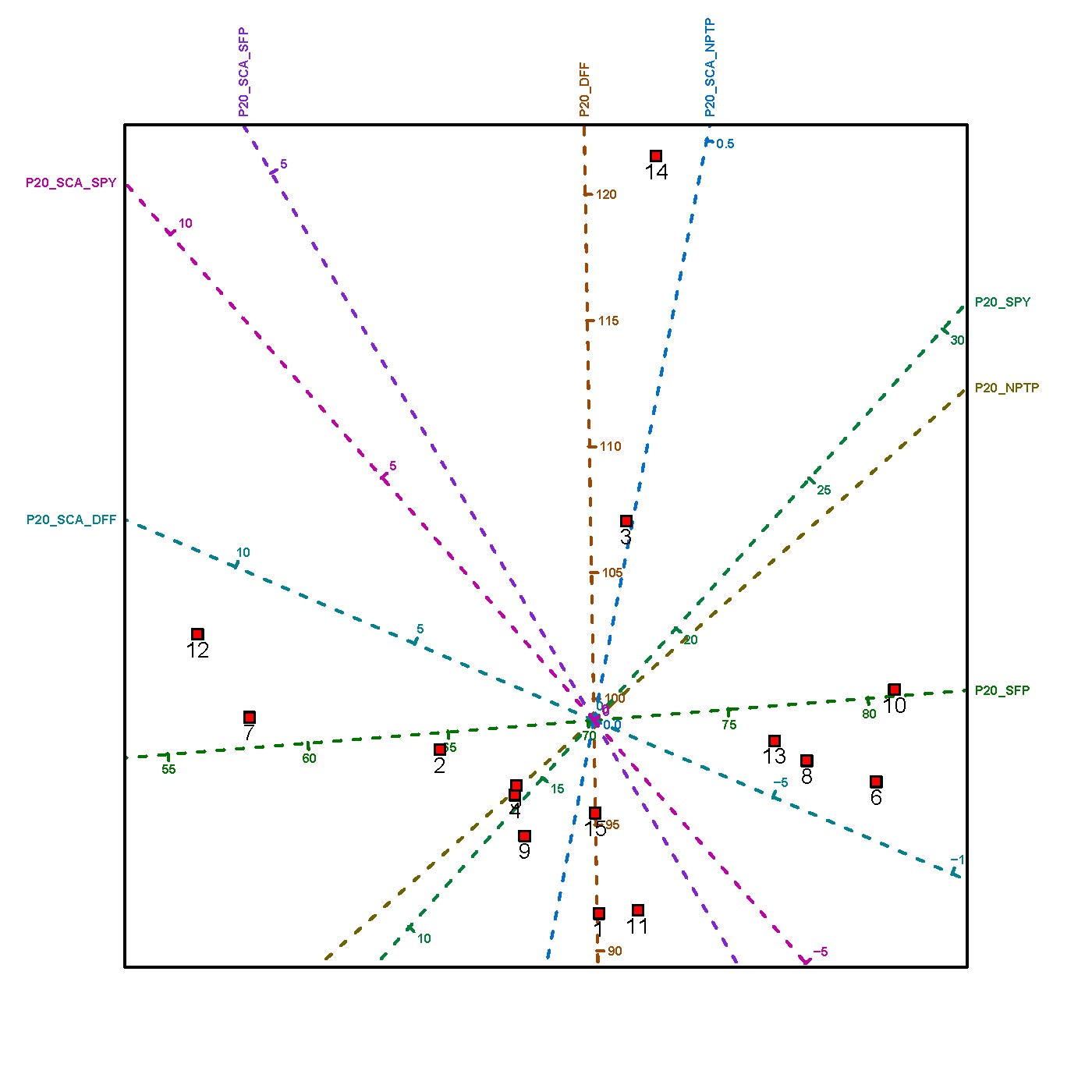


**Figure S5.** Principal component analysis (PCA) biplot diagrams for crosses representing Specific Combining Ability (SCA) and mean values of traits in P 40 kg ha^-1^. 1 to 15 indicates the hybrids H1 to H15.


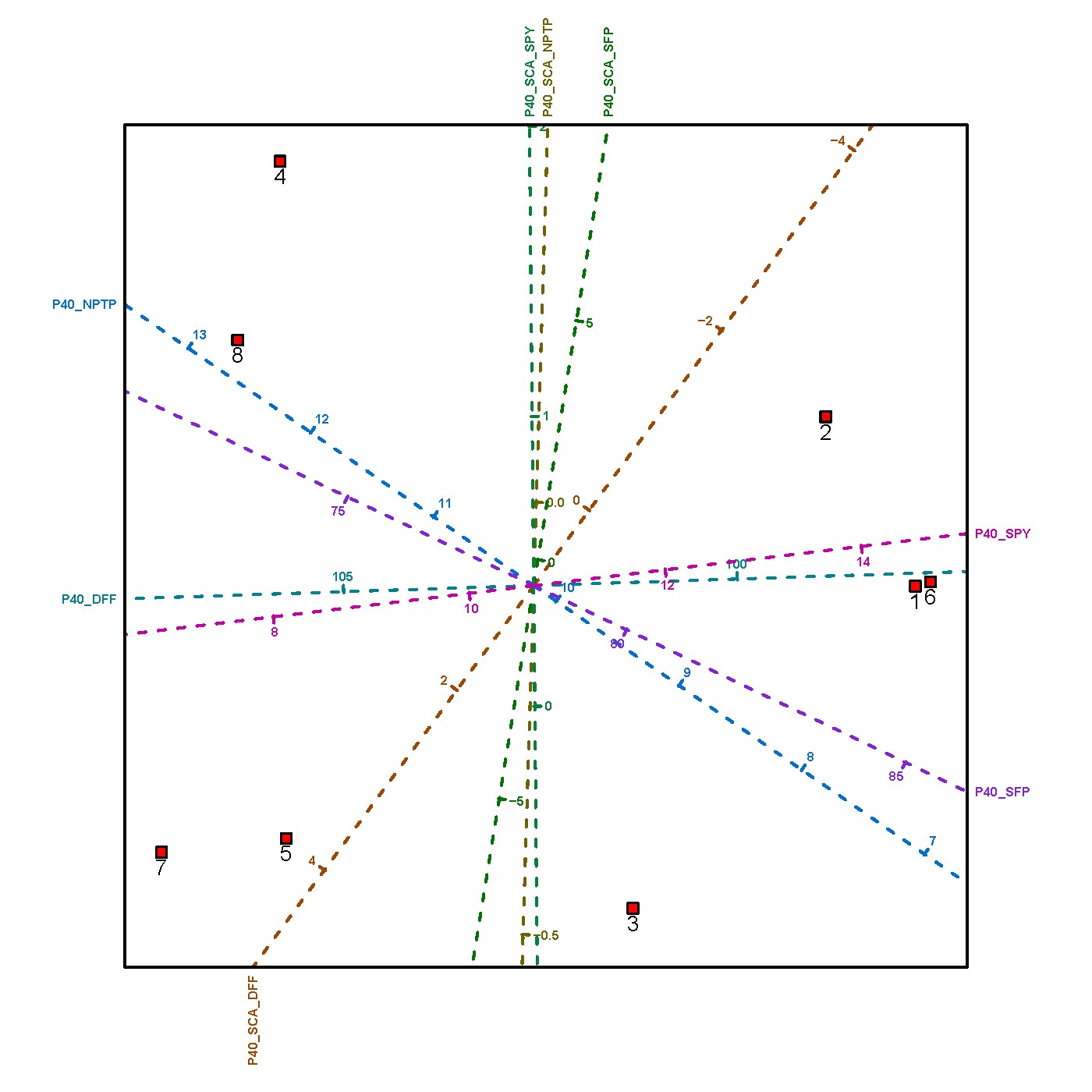


**Figure S6.** Principal component analysis (PCA) biplot diagrams for crosses representing Specific Combining Ability (SCA) and mean values of traits in P 60 kg ha^-1^. 1 to 15 indicates the hybrids H1 to H15.


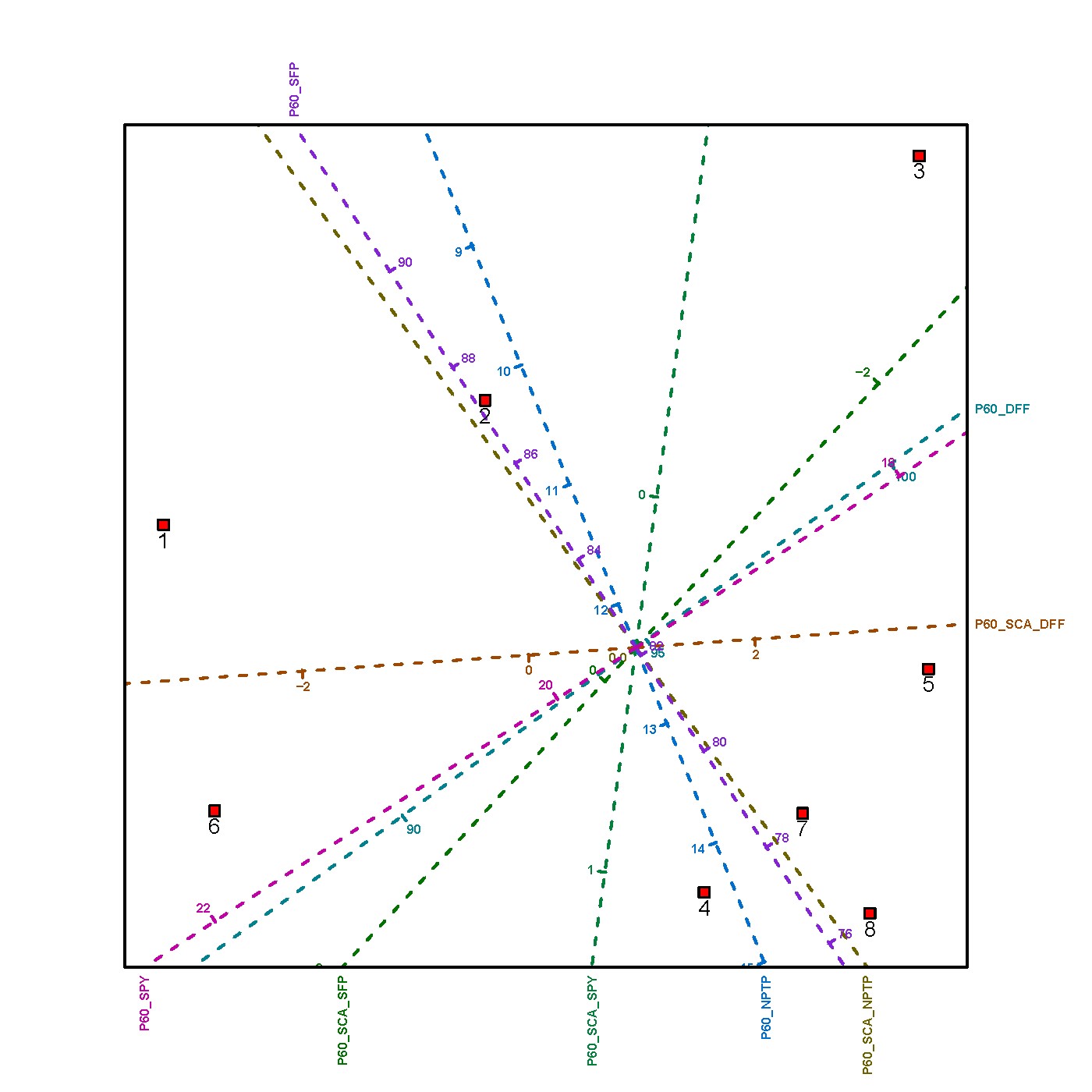


**Figure S7.** Principal component analysis (PCA) biplot diagrams for crosses representing Mid-Parent Heterosis (MPH) and mean values of traits in P 20 kg ha^-1^. 1 to 15 indicates the hybrids H1 to H15.


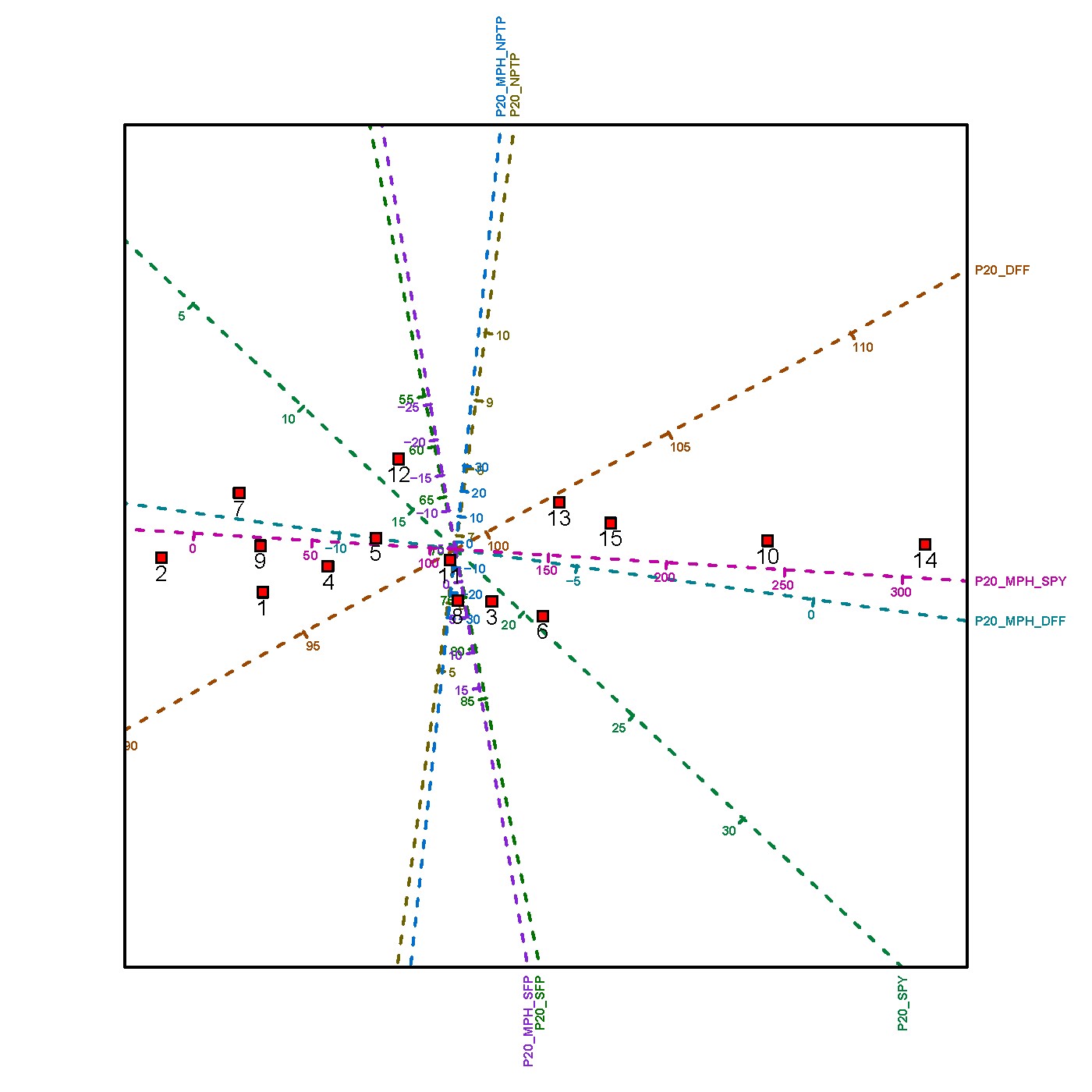


**Figure S8.** Principal component analysis (PCA) biplot diagrams for crosses representing Mid-Parent Heterosis (MPH) and mean values of traits in P 40 kg ha^-1^. 1 to 15 indicates the hybrids H1 to H15.


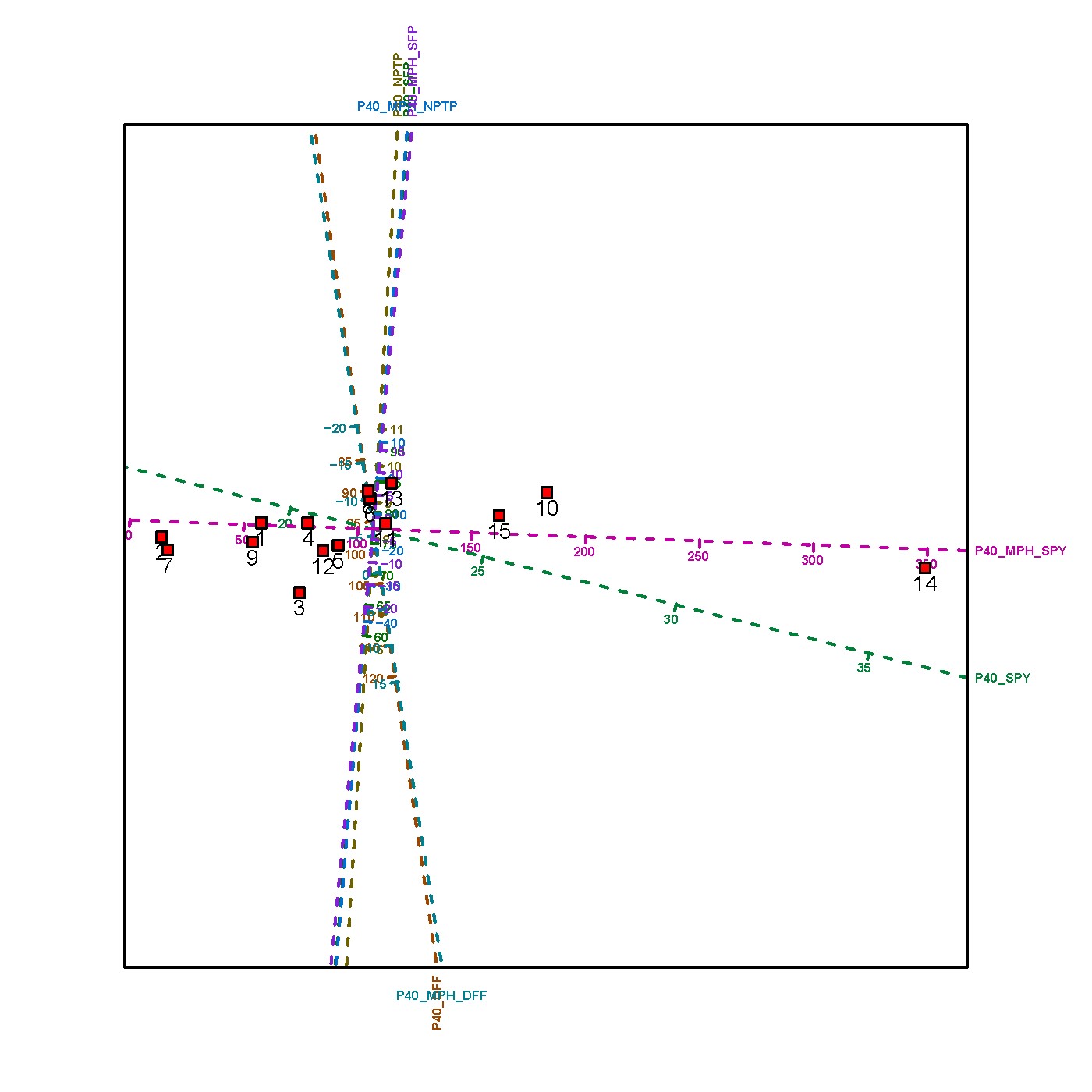


**Figure S9.** Principal component analysis (PCA) biplot diagrams for crosses representing Mid-Parent Heterosis (MPH) and mean values of traits in P 60 kg ha^-1^. 1 to 15 indicates the hybrids H1 to H15.


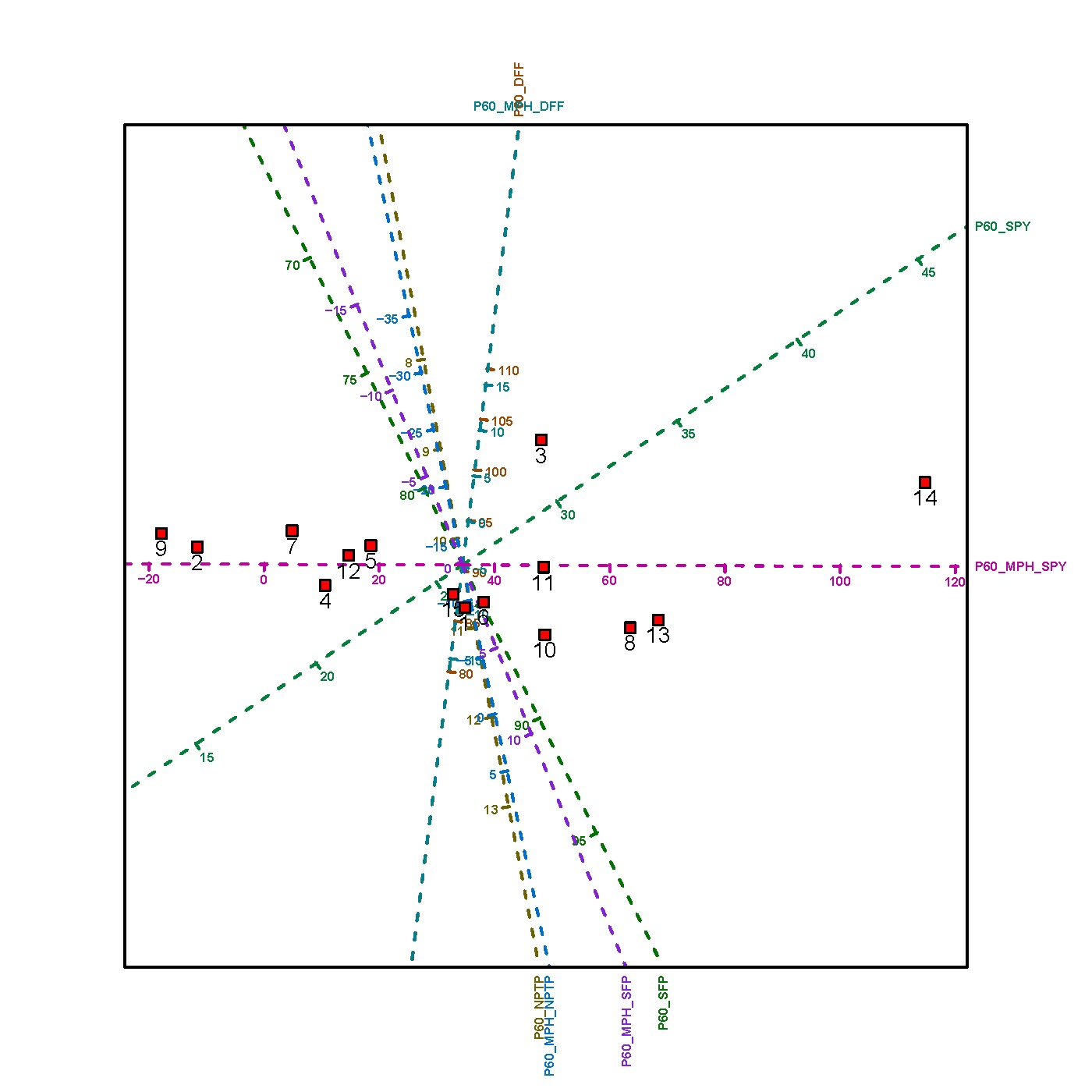

Supplement: Supplementary file 1 — Supplementary Material 1 [file 41598_2025_20344_MOESM1_ESM.docx]
